# An Analysis of the Timeline to Diagnosis and Treatment in Oral Cavity and Oropharynx Cancer

**DOI:** 10.1111/odi.70171

**Published:** 2025-12-26

**Authors:** Josefina Martínez‐Ramírez, Thaís Bianca Brandão, Carolina Guimarães Bonfim Alves, Letícia Rodrigues‐Oliveira, Luiz Paulo Kowalski, Tatiana Natasha Toporcov, Alan Roger Santos‐Silva, Ana Carolina Prado Ribeiro

**Affiliations:** ^1^ Oral Diagnosis Department University of Campinas Piracicaba Brazil; ^2^ Faculty of Dentistry University of El Salvador San Salvador El Salvador; ^3^ Dental Oncology Service, São Paulo State Cancer Institute São Paulo Brazil; ^4^ Department of Head and Neck Surgery University of São Paulo Medical School São Paulo Brazil; ^5^ Department of Epidemiology School of Public Health, University of São Paulo São Paulo Brazil

**Keywords:** delayed diagnosis, diagnostic itinerary, diagnostic journey, early detection, oral cavity cancer, oropharynx cancer

## Abstract

**Objective:**

To estimate the time intervals from first symptom to treatment initiation and to explore the multifaceted challenges faced by patients with oral cavity and oropharynx squamous cell carcinoma (OCSCC and OPSCC) during their diagnostic journey.

**Methods:**

A cross‐sectional study was conducted with 182 patients diagnosed with OCSCC or OPSCC admitted at the São Paulo State Cancer Institute between January 2019 and November 2020. The patient interval (PI), health system diagnostic interval (HSDI), and pretreatment interval (PTI) were evaluated.

**Results:**

Most patients were diagnosed at stage T3/T4 for both OCSCC (74.1%) and OPSCC (77.4%). The median PI was 1 month (IQR: 0–3), the median HSDI was 3 months (IQR: 2–6), and the median PTI was 3 months (IQR: 2–4). Patients with p16‐positive OPSCC were slightly more likely to experience a longer PI (IRR = 2.38; 95% CI: 1.00–5.67; *p* = 0.0499). The number of healthcare services visited showed the strongest association with progressively increased HSDI.

**Conclusions:**

Sociodemographic and clinical factors were associated with variations in the duration of the interval from symptom onset to treatment initiation. Targeted interventions to streamline referral pathways, strengthen professional training, and increase awareness among high‐risk populations could substantially improve timeliness of care and patient outcomes.

## 1 Introduction

Oral cavity and oropharynx squamous cell carcinoma (OCSCC and OPSCC) are among the most common cancers worldwide, accounting for an estimated 463,018 new cases in individuals over 40 years of age across both sexes in 2022 (Ferlay et al. 2024). These malignancies continue to represent a substantial global health burden, with delayed diagnosis contributing significantly to poor outcomes and high mortality rates (IARC 2023). The causes of diagnostic delay are multifactorial but are often linked to limited knowledge about the disease among at‐risk populations and primary care practitioners (Warnakulasuriya and Greenspan 2020).

Healthcare disparities (Figueiredo Lebre Martins et al. 2024), restricted access to healthcare facilities (Martínez‐Ramírez et al. 2024), and socioeconomic barriers further limit timely consultations with health professionals (Freire et al. 2021). Within medical and dental systems, providers may struggle to recognize the subtle signs and symptoms of early‐stage oral cancer, which are frequently asymptomatic and less clinically apparent. Such challenges contribute to delays in referral to specialists and prolonged intervals before definitive diagnosis is achieved (Langton et al. 2020).

Emerging diagnostic technologies, including adjunctive imaging modalities, liquid biopsy, molecular biomarkers, and artificial intelligence models, hold promise for improving early detection and diagnostic accuracy. However, the integration of these approaches into routine clinical practice remains limited across many healthcare settings (Camalan et al. 2021; Carnielli et al. 2018; de Souza et al. 2023).

This cross‐sectional study was designed to estimate the time intervals from the first symptom to treatment initiation and to explore the multifaceted challenges faced by patients with OCSCC and OPSCC during their diagnostic journey.

## 2 Methods

### 2.1 Study Design

This cross‐sectional study included patients with OCSCC and OPSCC admitted for oncologic treatment at the São Paulo State Cancer Institute, São Paulo, Brazil, between January 2019 and November 2020. Ethical approval was obtained from the National Human Research Ethics Committee (CAAE: 99756718.8.0000.5418). The study was conducted in accordance with the principles of the Declaration of Helsinki and reported following the STROBE (Strengthening the Reporting of Observational Studies in Epidemiology) guidelines (Von Elm et al. 2007).

### 2.2 Participants

Convenience sampling was used based on patient availability during the recruitment period. Eligibility criteria included patients aged > 18 years with a histological diagnosis of squamous cell carcinoma of the oral cavity (ICD C02–C06) or oropharynx (ICD C01, C09, C10) within the 6 months prior to recruitment. Patients with recurrent or second primary OCSCC or OPSCC were excluded, as were those with verbal, physical, or cognitive impairments that hindered comprehension of the study or the ability to provide informed consent.

During the study period, 484 cancer patients were referred to the Dental Oncology Service for dental evaluation and treatment; of these, 182 (37.6%) met the eligibility criteria. Following the initial appointment at the Dental Oncology Service, eligible participants were invited to complete structured questionnaire–based data collection, as previously described by Alves et al. (2023), regarding their experiences throughout the diagnostic journey, from the first sign or symptom to cancer diagnosis. Written informed consent was obtained from all participants.

### 2.3 Data Collection

Data were collected using a structured questionnaire administered in private settings by two neutral, trained researchers (CGBA and LRO) who were not involved in participants' treatment. Each administration required 25–40 min per participant. All sessions were conducted in discreet, comfortable environments to ensure confidentiality, and the structured format was strictly followed to maintain standardization across participants (Appendix S1).

Clinical data, including cancer site, HPV status (p16 protein expression for OPSCC patients), cancer stage (AJCC 8th Edition), and oncologic treatment, were extracted from medical records using the Tasy System (HTML5, Philips Clinical Informatics, Blumenau, Brazil), a web‐based electronic medical record platform. All collected data were subsequently imported into a REDCap questionnaire (version 14.0.16, Vanderbilt University, Nashville, Tennessee, USA).

### 2.4 Questionnaire

A 15‐item questionnaire was developed, comprising two phases. Phase 1 collected sociodemographic data, including age, sex, ethnicity, marital status, education level, monthly income, and tobacco and alcohol use. Phase 2 addressed the diagnostic journey, with items on the first reported symptom and its location, the first healthcare professional consulted and date of consultation, the first healthcare service sought, the number of services visited before histopathological diagnosis, the professional who established the histopathological diagnosis, and the healthcare service where it was obtained (Appendix S1).

### 2.5 Estimation of Intervals

Three diagnostic intervals were calculated: the patient interval (PI), health system diagnostic interval (HSDI), and pretreatment interval (PTI). PI was defined as the time from symptom onset to the first healthcare professional visit; HSDI as the time from the first healthcare professional visit to histopathological diagnosis; and PTI as the time from histopathological diagnosis to the initiation of oncologic treatment. All time points and intervals were measured in accordance with the Aarhus statement (Weller et al. 2012).

### 2.6 Statistical Analysis

**FIGURE 1:**
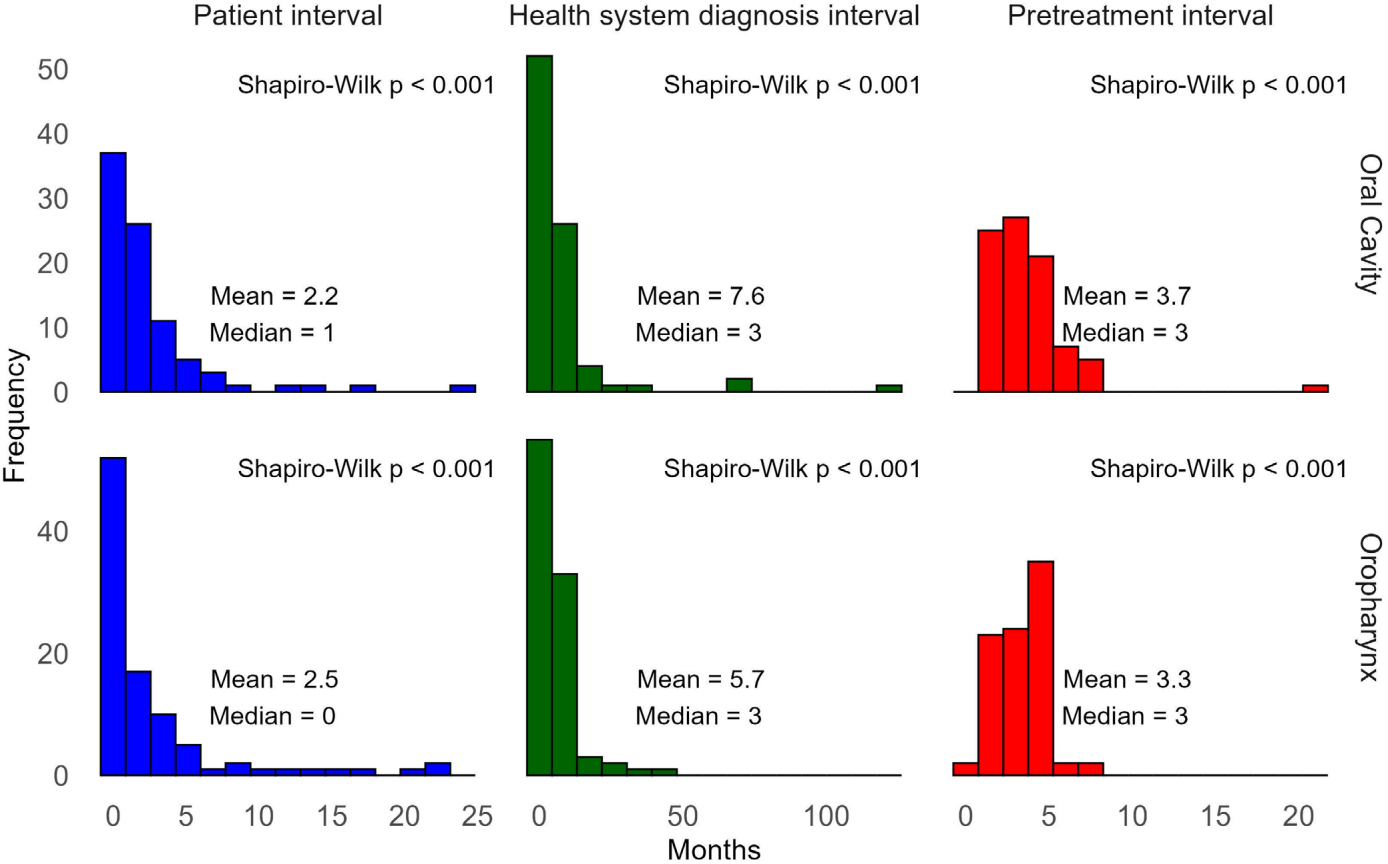
Histograms of time intervals in the diagnostic journey for oral (top row) and oropharynx (bottom row) squamous cell carcinoma. Patient intervals (blue), health system diagnostic intervals (green), and pretreatment intervals (red) all showed significant skewness (Shapiro–Wilk test, *p* < 0.001), with median values consistently lower than the means.

All analyses were performed using R software (version 4.4.3). Interval distributions were visually inspected with histograms stratified by primary tumor site (Figure 1). The Shapiro–Wilk test indicated non‐normality (*p* < 0.001), justifying the use of medians and interquartile ranges (IQR) for descriptive statistics and the application of non‐parametric tests for group comparisons.

As an exploratory step, univariate Cox proportional hazards models were fitted for each predictor and for each of the three intervals, treating time as a continuous variable in months. Hazard ratios (HR) and 95% confidence intervals (CI) were reported, using the most frequent category as the reference (Tables S1–, S3).

Given the skewness and overdispersion observed in the interval data (variance exceeding the mean), negative binomial regression (NB) was selected as the primary multivariable modeling strategy. In this approach, time (in months) was modeled as count data, and results were expressed as incidence rate ratios (IRR) with 95% CI. Variables with *p* < 0.20 in the univariate Cox models, along with those considered clinically relevant regardless of statistical significance, were included in the NB models to ensure comprehensive adjustment for potential confounding. Multicollinearity was assessed using the variance inflation factor (VIF), with values < 5 indicating no significant collinearity. For each adjusted model, coefficients, standard errors, IRR, and *p*‐values were reported (Tables S4–, S9).

## 3 Results

### 3.1 Sample Characteristics

A total of 87 patients with OCSCC and 95 with OPSCC were included. The majority were male (80.8%), and 63.7% were between 41 and 60 years of age at diagnosis. Most patients presented with advanced disease (stage III/IV) for both OCSCC (83.9%) and OPSCC (85.3%) (Table S10). Regarding tumor size, the majority were diagnosed with T3/T4 lesions in both OCSCC (74.1%) and OPSCC (77.4%).

Symptom profiles differed by tumor site: patients with OPSCC most frequently reported pain (41.1%) and lumps (28.4%), whereas those with OCSCC predominantly reported ulcers (49.9%) and pain (21.8%). Initial healthcare‐seeking behavior also varied. Patients with OPSCC most often sought care at public primary care centers (48.4%), followed by private services (26.3%). In contrast, patients with OCSCC more commonly sought care in private services (42.5%), with 31.0% first accessing public healthcare services (Table S11).

### 3.2 Patient Interval

The median PI was 1 month (IQR: 0–3) for OCSCC and 0 months (IQR: 0–3) for OPSCC. In OCSCC, shorter PI was associated with Black race (IRR = 0.23; 95% CI: 0.08–0.73; *p* = 0.012). These findings should be interpreted with caution, as most Black patients were still diagnosed at advanced stages (91.7%), similar to other racial groups (Table S12). Shorter PI was also associated with N1 lymph node involvement (IRR = 0.23; 95% CI: 0.07–0.81; *p* = 0.022) and with visiting three healthcare services prior to diagnosis (IRR = 0.32; 95% CI: 0.11–0.96; *p* = 0.042).

For OPSCC, shorter PI was linked to monthly income above one minimum wage (IRR = 0.42; 95% CI: 0.19–0.97; *p* = 0.042) and to visiting three (IRR = 0.17; 95% CI: 0.05–0.61; *p* = 0.006), four (IRR = 0.12; 95% CI: 0.02–0.57; *p* = 0.008), or six healthcare services (IRR = 0.04; 95% CI: 0.00–0.70; *p* = 0.027) before diagnosis. Conversely, mixed race (IRR = 2.25; 95% CI: 1.02–4.99; *p* = 0.046) and p16‐positive status (IRR = 2.38; 95% CI: 1.00–5.67; *p* = 0.049) were associated with longer PI (Table 1).

**TABLE 1:** Median intervals and significant predictors in multivariable negative binomial regression, stratified by primary tumor site.

Interval	Primary tumor site	Median (IQR)	Category	Variable	IRR	95% CI	*p* value
Patient interval	OCSCC	1 (0–3)	Race	Black	0.23	(0.08–0.73)	0.0120*
			N—Lymph node involvement	N1	0.23	(0.07–0.81)	0.0220*
			Number of services visited until diagnosis	3	0.32	(0.11–0.96)	0.0422*
	OPSCC	0 (0–3)	Monthly income	> 1 minimum wage	0.42	(0.19–0.97)	0.0420*
			Race	Mixed	2.25	(1.02–4.99)	0.0458*
			P16	Positive	2.38	(1.00–5.67)	0.0499*
			Number of services visited until diagnosis	3	0.17	(0.05–0.61)	0.0065**
				4	0.12	(0.02–0.57)	0.0080**
				6	0.04	(0.00–0.70)	0.0266*
	Total	1 (0–3)					
Health system diagnosis interval	OCSCC	3 (1.5–6)	Etilism	Yes/Former drinker	0.59	(0.36–0.99)	0.0450*
			Clinical staging	III	0.18	(0.07–0.47)	< 0.001***
				IV	0.22	(0.10–0.52)	< 0.001***
			Number of services visited until diagnosis	2	6.12	(2.47–15.18)	< 0.001***
				3	4.11	(1.60–10.56)	0.0033**
				4	4.34	(1.54–12.22)	0.0055**
				5	10.92	(3.69–32.31)	< 0.001***
	OPSCC	3 (2–6.5)	Marital status	Windowed	2.40	(1.39–4.26)	0.0021**
			Education (years of schooling)	1–3	0.43	(0.23–0.81)	0.0090**
				4–7	0.53	(0.33–0.85)	0.0076**
				15 or more	0.28	(0.14–0.55)	< 0.001***
			Number of services visited until diagnosis	2	2.55	(1.21–5.60)	0.0142*
				3	3.29	(1.61–7.00)	0.0012**
				4	3.17	(1.46–7.16)	0.0038**
				5	3.26	(1.33–8.29)	0.0109*
				6	8.23	(3.35–21.46)	< 0.001***
	Total	3 (2–6)					
Pretreatment interval	OCSCC	3 (2–3)	Marital status	Widowed	2.35	(1.41–3.92)	0.0010**
	OPSCC	3 (2–4)	None	None	None		
	Total	3 (2–4)					

*Note:* Statistical significance is indicated by the following codes: ***p < 0.001; **p < 0.01; *p < 0.05.

### 3.3 Health System Diagnosis Interval

The median HSDI was 3 months for both OCSCC (IQR: 1.5–6) and OPSCC (IQR: 2–6.5). In OCSCC, shorter HSDI was associated with alcohol consumption (IRR = 0.59; 95% CI: 0.36–0.99; *p* = 0.045) and advanced clinical stage (Stage III: IRR = 0.18; 95% CI: 0.07–0.47; *p* < 0.001; Stage IV: IRR = 0.22; 95% CI: 0.10–0.52; *p* < 0.001).

The number of healthcare services visited before diagnosis was a consistent factor influencing HSDI. Compared with patients visiting only one service, those visiting two experienced a 512% increase in time (IRR = 6.12; 95% CI: 2.47–15.18; *p* < 0.001), three services a 311% increase (IRR = 4.11; 95% CI: 1.60–10.56; *p* = 0.0033), four services a 334% increase (IRR = 4.34; 95% CI: 1.54–12.22; *p* = 0.0055), and five services a 992% increase (IRR = 10.92; 95% CI: 3.69–32.31; *p* < 0.001).

For OPSCC, the number of services visited was also directly associated with progressively longer HSDI, with IRR values of 2.55 for two services (*p* = 0.014), 3.29 for three (*p* = 0.001), 3.17 for four (*p* = 0.004), 3.26 for five (*p* = 0.011), and 8.23 for six services (*p* < 0.001) (Table 1).

### 3.4 Pretreatment Interval

For PTI, data were available for 86 patients with OCSCC and 88 with OPSCC, as eight patients died before initiating cancer treatment. The median PTI was 3 months for both tumor sites (OCSCC: IQR 2–3; OPSCC: IQR 2–4). In OCSCC, widowed status was associated with longer PTI (IRR = 2.35; 95% CI: 1.41–3.92; *p* = 0.001). No significant predictors of PTI were identified for OPSCC (Table 1).

## 4 Discussion

The results of this study highlight the heterogeneity in the duration of diagnostic and treatment intervals, which varied according to patients' sociodemographic and clinical characteristics. In all intervals analyzed, the median values were noticeably lower than the apparent means, underscoring the skewed distribution of these variables. Notably, patients with p16‐positive OPSCC were slightly more likely to experience longer intervals between symptom onset and medical consultation than p16‐negative patients, while widowed status was associated with more prolonged HSDI and PTI. Across tumor sites, the number of healthcare services visited before diagnosis emerged as the strongest factor associated with progressively longer HSDI.

Prolonged diagnostic intervals—shaped by both patient‐ and system‐related factors—are known to contribute to advanced‐stage presentation and poorer outcomes (Fernández‐Martínez et al. 2023; Gómez et al. 2009). Consistent with this, most patients in our study were diagnosed at advanced stages for both OCSCC and OPSCC. Epidemiological studies in Brazil similarly report high rates of late‐stage diagnosis (Abrahão et al. 2020; Da Costa et al. 2023; Kowalski et al. 2020; Louredo et al. 2022; Zavarez et al. 2020), with survival outcomes particularly unfavorable in regions with lower social indices (Louredo et al. 2022; de Pereira et al. 2023).

The PI is influenced by multiple factors, including the perception of symptoms as non‐urgent, limited awareness of HPV, low oral health literacy, socioeconomic barriers, fear, cultural beliefs, reliance on alternative medicine, and the role of initial healthcare consultants. These collectively contribute to delays in seeking specialized care (Al‐Kaabi et al. 2016; Mauceri et al. 2022; Noonan 2014; Queenan et al. 2018; Varela‐Centelles et al. 2018). We observed a median PI of 1 month, which aligns with pooled estimates for upper‐middle‐income countries (36 days; 95% CI: 21–38) and high‐income countries (31 days; 95% CI: 22–87) (Fernández‐Martínez et al. 2023). Shorter PI in OCSCC was associated with Black race and N1 lymph node involvement, whereas in OPSCC, mixed race and p16‐positive tumors were associated with longer PI. Previous studies indicate that limited symptom recognition and misinterpretation of early signs are particularly common among socioeconomically disadvantaged populations (Freire et al. 2021; Menezes et al. 2023; Póvoa et al. 2025). Furthermore, p16‐positive tumors often present atypically, commonly as a neck mass with less frequent odynophagia, which may contribute to delays in seeking care (McGarey Jr et al. 2024). In Brazil, these challenges are exacerbated by structural, socioeconomic, and cultural barriers (Figueiredo Lebre Martins et al. 2024), including stigma and limited healthcare access, which discourage timely consultation (Da Costa et al. 2023; Martínez‐Ramírez et al. 2024; Póvoa et al. 2025).

Among the three intervals, the HSDI was the longest and most variable, indicating that system‐related delays are the major contributor to overall time to treatment. In OCSCC, shorter HSDI was associated with alcohol consumption and advanced disease stage, likely reflecting expedited referral when disease is clinically evident. In contrast, visiting multiple services before diagnosis was strongly associated with longer HSDI, with incidence rate ratios exceeding 10 when five services were visited. In OPSCC, higher education was associated with shorter HSDI, while widowed status and visiting multiple services were linked to prolonged intervals. These findings underscore the cumulative burden of repeated consultations, including financial costs, emotional strain, and navigation challenges in fragmented care systems (Coxon et al. 2018). Similar associations between multiple pre‐referral consultations and diagnostic delays have been reported in Spain (Varela‐Centelles et al. 2021) and Brazil, where professional delays were linked to advanced‐stage diagnosis (Carvalho et al. 2002; Kowalski et al. 1994). Previous research has shown that insufficient knowledge of early warning signs among healthcare professionals contributes to misdiagnosis, inappropriate prescriptions, and unnecessary procedures that prolong referral to specialists (Carrard et al. 2018; Da Costa et al. 2023; Lima et al. 2021; Mauceri et al. 2022; Strey et al. 2022). Consistently, our study found shorter diagnostic times among patients with advanced‐stage disease, reflecting the difficulty of diagnosing early‐stage tumors, particularly in the context of limited awareness among both providers and the general population (Da Costa et al. 2023; Mauceri et al. 2022; Robb et al. 2009).

In Brazil, delays in HSDI can also be attributed to structural challenges, such as a shortage of specialists to perform biopsies and histopathology in socioeconomically disadvantaged regions, as well as fragmented referral systems (Da Costa et al. 2023; De‐Carli et al. 2023; Louredo et al. 2023; Martínez‐Ramírez et al. 2024). The literature lacks consensus on the threshold that defines a “delayed” diagnosis due to heterogeneous and arbitrarily selected time cutoffs. González‐Moles et al. (2022) propose defining late diagnosis at the point when tumors have progressed to stages associated with significant mortality risk and severe physical, psychological, and functional consequences. Tumor size may therefore serve as a more precise indicator of delayed diagnosis. Improving prognosis requires a comprehensive understanding of the actors, intervals, and determinants influencing the diagnostic process (González‐Moles et al. 2022; González‐Ruiz et al. 2023). Although Brazil's Unified Health System (SUS) guarantees universal coverage and free healthcare access, particularly benefiting low‐income populations (Ortega and Pele 2023), regional disparities persist, leading to inequities in access and quality of care (Chiliti et al. 2022; de Paiva et al. 2024; de Pereira et al. 2023; Póvoa et al. 2025; Raymundo et al. 2022). Similar structural challenges have been described in other low‐ and middle‐income regions, including Latin America, the Caribbean (Barrios et al. 2021), and India (Rath et al. 2018).

This study has several strengths, including the use of standardized Aarhus definitions, stratified analysis by tumor site, and the application of negative binomial regression to address overdispersed and asymmetric data. The World Health Organization recognizes three fundamental intervals in early cancer diagnosis—awareness and care‐seeking (PI), diagnosis (HSDI), and treatment initiation (PTI) (World Health Organization 2017). Our analysis of these intervals shows that most patients reliant on public healthcare experienced prolonged HSDI, often consulting multiple providers before diagnosis. These findings highlight the urgent need to strengthen cancer care pathways through patient and professional education, clear referral networks, and integrated health programs (Da Costa et al. 2023; de Paiva et al. 2024; Felippu et al. 2016; Warnakulasuriya and Kerr 2021; Wee et al. 2016; Zavarez et al. 2020). Improving healthcare integration and streamlining referral systems are critical policy priorities (Amri et al. 2022; Collaço et al. 2024; Martínez‐Ramírez et al. 2024). Targeted strategies for cancer prevention and early diagnosis should be prioritized, especially in vulnerable populations, to address health disparities (Chiliti et al. 2022; Menezes et al. 2023; de Pereira et al. 2023). Long‐term monitoring of prevention strategies and public health policies will also be essential (de Anne Souza Alves França et al. 2025; World Health Organization 2017).

As detailed in Table 2, actionable measures span all three intervals (PI, HSDI, PTI). For decision‐makers, three immediate priorities emerge: (1) strengthen first‐contact detection with direct‐to‐biopsy/referral pathways; (2) reduce “service hopping” by clarifying referral maps and incorporating tele‐triage/teledentistry where appropriate; and (3) implement time‐to‐treatment monitoring with feedback at the network level. Progress can be tracked using proposed metrics, including oral cancer awareness indices, proportion diagnosed after ≤ 2 services, median HSDI/PTI in days, tumor size, and stage.

**TABLE 2:** Actionable recommendations to improve early diagnosis and treatment of OCSCC and OPSCC.

Interval	Actionable recommendations	Potential metrics of success
Patient interval	Launch targeted public health campaigns to raise awareness of common early OCSCC and OPSCC symptoms (white/red patches, ulcers, lumps, persistent pain) and risk factors (HPV, tobacco, alcohol) using accessible language and visuals	Increase in oral health literacy and public awareness of OCSCC and OPSCC symptoms
	Partner with community leaders and trusted local figures to address cultural stigma and promote early help‐seeking behavior	Increased engagement in OCSCC and OPSCC screening programs Improved attitudes toward OCSCC and OPSCC care
	Develop educational materials tailored to specific demographic groups, addressing common misconceptions about OCSCC and OPSCC	Increased utilization of available resources (e.g., social media) Improved health literacy scores
Healthcare Service Diagnostic Interval	Implement standardized training programs for primary care providers (physicians and dentists) to improve their ability to recognize early signs of OPSCC and perform thorough oral examinations	Increased referrals for suspected OCSCC and OPSCC Improved accuracy in identifying suspicious lesions
	Strengthen undergraduate curricula on oral cancer detection (physicians and dentists)	Increase opportunistic OCSCC and OPSCC screening Improved accuracy in identifying suspicious lesions
	Develop and implement integrated OPSCC care pathways that streamline the diagnostic, simplifying referral pathways within public healthcare systems to ensure timely access to specialist consultations and diagnostic procedures	Reduction in time from primary care visits to specialist consultation and diagnosis Improved patient navigation through the healthcare system
	Promote the use of teledentistry to improve access to specialist consultations in remote areas	Increased access to specialist consultations in remote areas Improved diagnosis rates
Pretreatment Interval	Implement strategies to reduce waiting times for cancer treatment, such as optimizing resource allocation and improving scheduling efficiency	**• R**eduction in waiting times for surgery, radiotherapy, and chemotherapy

*Note:* Regularly collect and monitor data on the duration of the three intervals to assess the effectiveness of the implemented measures in reducing their length.

This study also has limitations. First, the PI was based on patient‐reported data, which may be subject to recall bias, particularly for symptom onset and the timing of first consultations. Second, several analyzed subgroups yielded extreme hazard ratio values with wide confidence intervals due to small sample sizes, limiting robust conclusions in these categories. Third, the study period overlapped with the COVID‐19 pandemic, particularly between May and July 2020, when São Paulo experienced peak case volumes. Although the São Paulo State Cancer Institute remained operational throughout this period, ensuring continuity of oncologic care (Rodrigues‐Oliveira et al. 2022), disruptions in healthcare access may have occurred. Nonetheless, we believe that the prolonged intervals observed primarily reflect longstanding structural challenges in the healthcare system, including fragmented referral pathways, multiple consultations (Carvalho et al. 2002; Kowalski et al. 1994), and limited awareness among both the public and healthcare professionals.

Future research should aim to validate and expand these findings through multicenter studies, enabling a deeper understanding of the patient, provider, and system‐level factors influencing diagnostic and treatment intervals in OCSCC and OPSCC. Ongoing monitoring of prevention programs and the integration of advanced diagnostic tools will also be crucial to improving early detection and outcomes.

## 5 Conclusion

This study demonstrates the heterogeneity in diagnostic and treatment intervals, with sociodemographic and clinical factors influencing the time from symptom onset to treatment initiation. The health system diagnostic interval emerged as the main contributor to delays in both OCSCC and OPSCC, with the number of consultations serving as the key driver of progressive prolongation. These findings underscore the need for targeted interventions to enhance early symptom recognition, streamline referral pathways, and improve diagnostic accuracy. Integrated public health policies, healthcare system reforms, and strengthened professional training are essential to reduce delays and promote earlier diagnosis.

## Author Contributions

**Josefina Martínez‐Ramírez:** data curation, formal analysis, validation, visualization, writing – original draft, writing – review and editing, investigation. **Thaís Bianca Brandão:** conceptualization, data curation, formal analysis, methodology, validation, supervision, visualization, writing – original draft, writing – review and editing, investigation. **Carolina Guimarães Bonfim Alves:** conceptualization, data curation, formal analysis, methodology, project administration, validation, supervision, visualization, writing – review and editing, investigation. **Letícia Rodrigues‐Oliveira:** project administration, writing – review and editing. **Luiz Paulo Kowalski:** writing – review and editing. **Tatiana Natasha Toporcov:** writing – review and editing, formal analysis. **Alan Roger Santos‐Silva:** conceptualization, investigation, methodology, project administration, supervision, visualization, writing – original draft, writing – review and editing. **Ana Carolina Prado Ribeiro:** conceptualization, writing – review and editing, project administration, methodology.

## Conflicts of Interest

The authors declare no conflicts of interest.

## Supporting information


**Data S1:** Supporting Information S1.

**Table S1:** Patient interval duration by sociodemographic and clinicopathological characteristics.

**Table S2:** Health system diagnostic interval duration by sociodemographic and clinicopathological characteristics.

**Table S3:** Pretreatment interval duration by sociodemographic, clinicopathological, and diagnostic journey characteristics.

**Table S4:** Negative binomial regression model of the patient interval in oral cavity cancer patients.

**Table S5:** Negative binomial regression model of the patient interval in oropharynx cancer patients.

**Table S6:** Negative binomial regression model of the health system diagnostic interval in oral cavity cancer patients.

**Table S7:** Negative binomial regression model of the health system diagnostic interval in oropharynx cancer patients.

**Table S8:** Negative binomial regression model of the pretreatment interval in oral cavity cancer patients.

**Table S9:** Negative binomial regression model of the pretreatment interval in oropharynx cancer patients.

**Table S10:** Sociodemographic and clinicopathological characteristics of patients by primary tumor site.

**Table S11:** Diagnostic itinerary characteristics by primary tumor site.

**Table S12:** Clinical stage by race.

## Data Availability

The data supporting the findings of this study are available from the corresponding author upon reasonable request. Data are not publicly available due to privacy and ethical restrictions.
