# Supplementary material for: An Analysis of the Timeline to Diagnosis and Treatment in Oral Cavity and Oropharynx Cancer
Source: Oral Dis. 2025 Dec 26;32(4):983–91. doi: 10.1111/odi.70171 (PMC13248584; doi:10.1111/odi.70171)
Supplement: Supplementary file 1 — Data S1: Supporting Information S1. [file ODI-32-983-s008.docx]

**ORIGINAL VERSION IN PORTUGUES**

**PROJETO DE PESQUISA: COMUNICAÇÃO DO DIAGNÓSTICO DE CÂNCER DE BOCA E OROFARINGE: AS PERCEPÇÕES DOS PACIENTES COM BASE NO PROTOCOLO SPIKES – ADENDO INVESTIGAÇÃO ITINERÁRIO TERAPEUTICO**

VOLUNTÁRIO: DATA TCLE:___/___/______

| CARACTERÍSTICAS SOCIODEMOGRÁFICAS |
| --- |
| Data de nascimento: ___/____/____ Gênero: [ ]Homem [ ]Mulher |
| Estado Civil: [ ]Solteiro [ ]Casado/vivendo com parceiro [ ]Divorciado/Separado [ ]Viúvo |
| Etnia: [ ]Branco [ ]Negro [ ]Pardo [ ]Amarelo [ ]Outro: ______________________ |
| Escolaridade (anos de estudo): [ ]Sem instrução ou <1 ano [ ]1 a 3 anos [ ] 4 a 7 anos [ ]8 a 10 anos  [ ]11 a 14 anos [ ]15 anos ou mais [ ] não informado |
| Renda: [ ]≤1 Salário mínimo [ ] > 1 Salário mínimo (salário mínimo SP R$1.108,38) |
| **CONTEXTO DA DESCOBERTA DO CÂNCER** |
| Primeiro sintoma percebido: [ ]Mancha [ ]Úlcera (ferida) [ ]Nódulo (caroço) [ ]Aumento de volume (inchaço) [ ]Dor   [ ] Ardência [ ] Sangramento [ ] Outro:____________________________________________ |
| Mês e ano do primeiro sintoma: ___/______ Local do primeiro sintoma: ____________________________________________ |
| Mês e ano em que procurou 1ª ajuda profissional: ___/______. |
| Qual profissional procurou: [ ]Médico [ ] Dentista [ ] Outro:_______________________________________________________ |
| Local em que procurou 1ª ajuda profissional: [ ]Serviço público [ ]Serviço privado. Especifique:  [ ]Consultório [ ]UBS/USF [ ]CEO/AME [ ]Hospital [ ]Pronto socorro [ ]Universidades [ ] Outro:_________ |
| Número de serviços/profissionais de saúde procurados até chegar ao diagnóstico histopatológico definitivo: _________________ |
| Local onde realizou diagnóstico definitivo: [ ]Serviço público [ ]Serviço privado. Especifique:  [ ]Consultório [ ]UBS/USF [ ]CEO/AME [ ]Hospital [ ]Pronto socorro [ ]Universidades [ ] Outro:_________ |
| Qual profissional realizou diagnóstico definitivo: [ ]Médico [ ]Dentista [ ] Outro:_____________________________________ |
| Data do diagnóstico histopatológico definitivo (conferir em prontuário)*: ____/____/________ |
| Observações: ______________________________________________________________________________________________  __________________________________________________________________________________________________________________________________________________________________________________________________________________ |
| **REVISÃO DE PRONTUÁRIO** |
| Data de admissão no serviço de tratamento: _____/__________ |
| Sítio do tumor: CID-10: ____________ Topografia:__________________________________ |
| Estadiamento Clínico: ______________ Patológico:____________ (TNM 8ªed) [ ]Sem registro |
| Status P16: [ ] positivo [ ]negativo [ ] não investigado [ ]sem registro |
| Data de início do tratamento oncológico: ______/________ Tipo: [ ] Curativo [ ] Paliativo |
| Modalidade: [ ] Cirurgia [ ]RT [ ]QT Adjuvancia: [ ] Cirurgia [ ]RT [ ]QT |
| **VARIÁVEIS CALCULADAS – INTERVALOS DE TEMPO** |
| 1. Primeiro sintoma >> Diagnóstico definitivo: _______________ (meses) – *“Total time to diagnosis -* ***TDI****”* |
| 2. Primeiro sintoma >> Primeira consulta profissional: _______________ (meses) – “*Patient interval -* ***PI***” |
| 3. Primeira consulta profissional >> Diagnóstico definitivo: _____________ (meses) – “D*iagnosis interval -* ***DI***” |
| 4. Diagnóstico definitivo >> Início do tratamento: ________________ (semanas) – “T*reatment interval -* ***TI***” |

**VERSION IN ENGLISH**

**PATIENT’S PERCEPTIONS OF ORAL AND OROPHARYNGEAL CANCER DIAGNOSIS DISCLOSURE: COMMUNICATION ASPECTS BASED ON SPIKES PROTOCOL. - ADDENDUM THERAPEUTIC ITINERARY RESEARCH**

VOLUNTEER: IC DATE:___/___/______

| SOCIODEMOGRAPHIC CHARACTERISTICS |
| --- |
| Date of birth: ___/____/____ Gender: [ ] Male [ ]Female |
| Marital status: [ ]Single [ ]Married/living with a partner [ ]Divorced/Separated [ ]Widowed |
| Ethnicity: [ ]White [ ]Black [ ]Mixed [ ]Asian [ ]Other: ______________________ |
| Education (years of study): [ ] No education or <1 year [ ]1 - 3 years [ ] 4 - 7 years [ ]8 - 10 years  [ ]11 - 14 years [ ]15 years or more [ ] No information |
| Monthly income: [ ]≤1 minimum wage [ ] > 1 minimum wage (minimum wage SP R$1.108,38) |
| **Cancer diagnosis itinerary** |
| Firts noticed symptom: [ ]Spot [ ]Ulcer (wound) [ ]Lump (mass) [ ]Swelling [ ]Pain   [ ]Burning [ ] Bleeding [ ] Other:____________________________________________ |
| Month and year of first symptom: ___/______ Site of the first symptom: ___________________________________________ |
| Month and years of the first healthcare service sought: ___/______. |
| First healthcare professional for evaluation: [ ]Physician [ ] Dentist [ ] Other:__________________________________________ |
| First healthcare service sought: [ ]Public service [ ]Private service. Specific:  [ ]Clinic [ ]UBS/USF [ ]CEO/AME [ ]Hospital [ ]Emergency room [ ]University dental clinic [ ] Other:_________ |
| Number of services visited until histopathological diagnosis: _________________ |
| Location of histopathological diagnosis: [ ]Public service [ ]Private service. Specific:  [ ]Clinic [ ]UBS/USF [ ]CEO/AME [ ]Hospital [ ]Emergency room [ ]University dental clinic [ ] Other:_________ |
| Professional delivering the histopathological diagnosis: [ ]Physician [ ]Dentist [ ] Other:____________________________ |
| Date of definitve histophatological diagnosis (check medical records)*: ____/____/________ |
| Comments: _______________________________________________________________________________________________  __________________________________________________________________________________________________________________________________________________________________________________________________________________ |
| **MEDICAL RECORDS** |
| Admission data to the treatment service: _____/__________ |
| Tumor site: CID-10: ____________ Topografia:__________________________________ |
| Clinical staging: ______________ Pathological: ____________ (TNM 8ªed) [ ]No record |
| P16 status: [ ] positive [ ]negative [ ]no analyzed [ ]no record |
| Date of oncological treatment start: ______/________ Type: [ ] Curative [ ] Palliative |
| Modality: [ ] Surgery [ ]RT [ ]QT Adjuvant: [ ] Surgery [ ]RT [ ]QT |
| **TIME POINTS – INTERVALS** |
| 1. First symptom >> Definitive diagnosis: _______________ (months) – *“Total time to diagnosis -* ***TDI****”* |
| 2. First symptom >> First healthcare professional visit: _______________ (months) – “*Patient interval -* ***PI***” |
| 3. First healthcare professional visit >> Definitive diagnosis: _____________ (months) – “D*iagnosis interval -* ***DI***” |
| 4. Definitive diagnosis >> Start treatment: ________________ (weeks) – “T*reatment interval -* ***TI***” |
