# Supplementary material for: An Analysis of the Timeline to Diagnosis and Treatment in Oral Cavity and Oropharynx Cancer
Source: Oral Dis. 2025 Dec 26;32(4):983–91. doi: 10.1111/odi.70171 (PMC13248584; doi:10.1111/odi.70171)
Supplement: Supplementary file 2 — Table S1: Patient interval duration by sociodemographic and clinicopathological characteristics. [file ODI-32-983-s013.docx]

**Table S1.** Patient interval duration by sociodemographic and clinicopathological characteristics.

| **Characteriscs** |  | **Oral cavity** |  |  |  | **Oropharynx** |  |  |
| --- | --- | --- | --- | --- | --- | --- | --- | --- |
|  | n | Median (IQR) | Hazard Ratio | P-value | n | Median (IQR) | Hazard Ratio | P-value |
| **Gender** |  |  |  |  |  |  |  |  |
| Male | 71 | 1 (0–2.5) | 1.00 | 0,803 | 76 | 0 (0–3) | 1.00 | 0,424 |
| Female | 16 | 1.5 (0–3) | 0.93 (0.54–1.61) |  | 19 | 0 (0–2) | 1.23 (0.74–2.05) |  |
| **Age at diagnosis** |  |  |  |  |  |  |  |  |
| ≤ 40 years | 5 | 1 (0–3) | 1.01 (0.4–2.54) |  | 2 | 2 (1–3) | 1.02 (0.25–4.2) |  |
| 41 - 60 years | 55 | 1 (0–2) | 1.00 | 0,981 | 61 | 0 (0–3) | 1.00 | 0,977 |
| > 60 years | 27 | 1 (0–3) | 1.02 (0.64–1.63) |  | 32 | 0 (0–2.25) | 1.14 (0.74–1.75) |  |
| **Ethnicity (self-reported)** |  |  |  |  |  |  |  |  |
| White | 33 | 1 (0–3) | 0.84 (0.53–1.33) |  | 33 | 0 (0–3) | 0.97 (0.62–1.53) |  |
| Black | 12 | 0.5 (0–1) | 1.65 (0.85–3.19) |  | 13 | 1 (0–3) | 0.93 (0.5–1.72) |  |
| Mixed | 42 | 1 (0–3) | 1.00 | 0,46 | 49 | 0 (0–1) | 1.00 | 0,909 |
| **Marital status** |  |  |  |  |  |  |  |  |
| Single | 22 | 1.5 (0–3) | 0.77 (0.45–1.3) |  | 29 | 0 (0–4) | 0.99 (0.62–1.61) |  |
| Married/living with a partner | 41 | 1 (0–2) | 1.00 | 0,323 | 45 | 1 (0–3) | 1.00 | 0,98 |
| Divorced/separated | 18 | 0 (0–1) | 1.31 (0.75–2.29) |  | 14 | 0 (0–0.75) | 1.29 (0.7–2.38) |  |
| Widowed | 6 | 0.5 (0–2.5) | 1.31 (0.55–3.11) |  | 7 | 0 (0–0.5) | 1.81 (0.81–4.04) |  |
| **Education** |  |  |  |  |  |  |  |  |
| < 1 year of schooling | 5 | 0 (0–1) | 1.33 (0.51–3.48) |  | 14 | 0 (0–0.75) | 1.34 (0.72–2.52) |  |
| 1 - 3 years of schooling | 18 | 1 (0–2.75) | 0.85 (0.47–1.55) |  | 12 | 0 (0–1.25) | 1.5 (0.77–2.93) |  |
| 4 - 7 years of schooling | 27 | 1 (0–1.5) | 1.00 | 0,555 | 34 | 0.5 (0–3) | 1.00 | 0,358 |
| 8 - 10 years of schooling | 11 | 2 (0–3) | 0.57 (0.28–1.19) |  | 12 | 0 (0–1) | 1.4 (0.72–2.73) |  |
| 11 - 14 years of schooling | 18 | 0.5 (0–1) | 0.84 (0.46–1.53) |  | 12 | 1.5 (0–5.25) | 0.84 (0.43–1.63) |  |
| 15 years of schooling or more | 8 | 4 (1–6.75) | 0.46 (0.21–1.03) |  | 11 | 1 (0–3) | 0.94 (0.47–1.87) |  |
| **Monthly income** |  |  |  |  |  |  |  |  |
| ≤ 1 minimum wage | 40 | 1 (0–2) | 1.27 (0.83–1.94) |  | 58 | 0.5 (0–3.75) | 1.00 | 0,093 |
| > 1 minimum wage | 47 | 1 (0–3) | 1.00 | 0,279 | 37 | 0 (0–1) | 1.44 (0.94–2.19) |  |
| **Smoking** |  |  |  |  |  |  |  |  |
| Never | 18 | 1 (0–3) | 0.78 (0.45–1.32) |  | 8 | 1 (0.75–3.5) | 0.87 (0.42–1.81) |  |
| Yes / Former smoker | 69 | 1 (0–2) | 1.00 | 0,352 | 87 | 0 (0–3) | 1.00 | 0,71 |
| **Alcohol comsumption** |  |  |  |  |  |  |  |  |
| Never | 21 | 1 (0–3) | 0.95 (0.58–1.56) |  | 16 | 0.5 (0–3.25) | 1.08 (0.62–1.86) |  |
| Yes / Former drinker | 66 | 1 (0–2) | 1.00 | 0,846 | 79 | 0 (0–3) | 1.00 | 0,789 |
| **Specific location of tumor** |  |  |  |  |  |  |  |  |
| Tongue | 42 | 1 (0–2) | 1.00 | 0,258 |  |  |  |  |
| Floor of mouth | 14 | 1 (0–3) | 0.7 (0.37–1.3) |  |  |  |  |  |
| Hard palate | 5 | 3 (3–9) | 0.39 (0.15–1.02) |  |  |  |  |  |
| Retromolar area | 11 | 0 (0–1) | 1.29 (0.66–2.51) |  |  |  |  |  |
| Alveolar ridge | 6 | 0.5 (0–2.5) | 1.05 (0.44–2.47) |  |  |  |  |  |
| Gengiva | 3 | 1 (0.5–6.5) | 0.54 (0.16–1.79) |  |  |  |  |  |
| Buccal mucosa | 6 | 1.5 (0.25–2.75) | 0.48 (0.18–1.25) |  |  |  |  |  |
| **p16 status** |  |  |  |  |  |  |  |  |
| Negative | 19 | 1 (0–3) | 0.96 (0.57–1.6) |  | 72 | 0 (0–1.25) | 1.00 | 0,264 |
| Positive | 6 | 5 (1–6) | 0.63 (0.27–1.49) |  | 23 | 1 (0–4) | 0.76 (0.48–1.23) |  |
| Unknown | 62 | 1 (0–2) | 1.00 | 0,863 |  |  |  |  |
| **T – Tumor size** |  |  |  |  |  |  |  |  |
| T1 | 8 | 0.5 (0–2.25) | 1.07 (0.5–2.28) |  | 9 | 1 (0–4) | 0.89 (0.43–1.86) |  |
| T2 | 14 | 0.5 (0–2.75) | 1.17 (0.64–2.14) |  | 12 | 0 (0–0.25) | 1.58 (0.82–3.07) |  |
| T3 | 17 | 1 (0–2) | 0.99 (0.56–1.76) |  | 31 | 1 (0–3) | 1.09 (0.67–1.78) |  |
| T4 | 46 | 1 (0–3) | 1.00 | 0,856 | 41 | 0 (0–2) | 1.00 | 0,764 |
| Tx | 2 | 1.5 (1.25–1.75) | 1.06 (0.26–4.42) |  | 2 | 1 (0.5–1.5) | 1.36 (0.33–5.71) |  |
| **N – Lymph node involvement** |  |  |  |  |  |  |  |  |
| N0 | 28 | 1 (0–3) | 1.00 | **0,021** | 12 | 0 (0–1.25) | 1.36 (0.7–2.66) |  |
| N1 | 10 | 0 (0–0.75) | 2.41 (1.14–5.09) |  | 21 | 0 (0–3) | 1.04 (0.6–1.81) |  |
| N2 | 27 | 1 (0–4) | 1.07 (0.62–1.82) |  | 33 | 0 (0–2) | 1.00 | 0,367 |
| N3 | 22 | 1 (0–2) | 1.52 (0.86–2.71) |  | 29 | 1 (0–4) | 0.87 (0.52–1.44) |  |
| **M – distant metastasis** |  |  |  |  |  |  |  |  |
| M0 | 85 | 1 (0–3) | 1.00 | 0,252 | 92 | 0 (0–3) | 1.00 | 0,971 |
| M1 | 2 | 7.5 (4.25–10.75) | 0.44 (0.1–1.8) |  | 3 | 0 (0–4) | 0.98 (0.31–3.11) |  |
| **Clinical staging** |  |  |  |  |  |  |  |  |
| I | 7 | 1 (0–2.5) | 0.87 (0.4–1.91) |  | 5 | 0 (0–0) | 1.2 (0.48–3) |  |
| II | 7 | 3 (0–4.5) | 0.75 (0.34–1.66) |  | 9 | 0 (0–3) | 0.94 (0.46–1.91) |  |
| III | 14 | 1 (0–2.5) | 0.78 (0.42–1.43) |  | 24 | 1.5 (0–4) | 0.68 (0.42–1.11) |  |
| IV | 59 | 1 (0–2) | 1.00 | 0,726 | 57 | 0 (0–1) | 1.00 | 0,701 |
| **Location of first symptom** |  |  |  |  |  |  |  |  |
| Oral cavity | 72 | 1 (0–3) | 1.00 | 0,061 | 15 | 1 (0–2) | 1.1 (0.6–2.01) |  |
| Cervical region | 2 | 0 (0–0) | 3.94 (0.94–16.57) |  | 34 | 0 (0–2.75) | 1.16 (0.72–1.85) |  |
| Oropharynx | 5 | 3 (2–6) | 0.66 (0.27–1.65) |  | 38 | 1 (0–3) | 1.00 | 0,767 |
| Others | 8 | 0.5 (0–3) | 0.85 (0.4–1.79) |  | 8 | 0 (0–0) | 2.37 (1.08–5.16) |  |
| **First noticed symptom by topography** |  |  |  |  |  |  |  |  |
| **Oral cavity** |  |  |  |  |  |  |  |  |
| Ulcer (wound) | 43 | 1 (0–2.5) | 1.00 | 0,55 | 10 | 0.5 (0–1) | 1.08 (0.52–2.24) |  |
| Pain | 7 | 1 (0.5–1.5) | 1.28 (0.57–2.88) |  | 3 | 0 (0–0.5) | 1.97 (0.59–6.59) |  |
| Lump (mass) | 6 | 2 (0.25–3) | 0.83 (0.35–1.96) |  | 1 | 12 (12–12) | 0.32 (0.04–2.39) |  |
| Spot | 8 | 1 (0–6) | 0.69 (0.32–1.49) |  |  |  |  |  |
| Swelling | 3 | 0 (0–0.5) | 2.44 (0.74–8.02) |  | 1 | 5 (5–5) | 0.48 (0.06–3.58) |  |
| Bleeding | 2 | 3 (2–4) | 0.7 (0.17–2.92) |  |  |  |  |  |
| Others | 3 | 0 (0–0.5) | 2.44 (0.74–8.02) |  |  |  |  |  |
| **Oropharynx** |  |  |  |  |  |  |  |  |
| Pain | 4 | 2.5 (1.5–3.75) | 0.75 (0.27–2.1) |  | 29 | 0 (0–2) | 1.00 | 0,269 |
| Spot | 1 | 6 (6–6) | 0.42 (0.06–3.09) |  | 3 | 3 (1.5–5) | 0.7 (0.21–2.32) |  |
| Ulcer (wound) |  |  |  |  | 2 | 6 (5–7) | 0.44 (0.1–1.88) |  |
| Lump (mass) |  |  |  |  | 1 | 0 (0–0) | 3.35 (0.45–25.08) |  |
| Swelling |  |  |  |  | 1 | 1 (1–1) | 1.08 (0.15–8.03) |  |
| Others |  |  |  |  | 2 | 8 (6.5–9.5) | 0.4 (0.09–1.7) |  |
| **Cervical region** |  |  |  |  |  |  |  |  |
| Pain | 1 | 0 (0–0) | 4.21 (0.57–31.37) |  | 4 | 0 (0–0) | 3.35 (1.14–9.88) |  |
| Lump (mass) |  |  |  |  | 24 | 0.5 (0–4.5) | 0.81 (0.46–1.41) |  |
| Swelling |  |  |  |  | 6 | 0 (0–0.75) | 1.79 (0.73–4.4) |  |
| Others | 1 | 0 (0–0) | 4.21 (0.57–31.37) |  |  |  |  |  |
| **Others** |  |  |  |  |  |  |  |  |
| Pain | 7 | 0 (0–5) | 0.78 (0.34–1.77) |  | 3 | 0 (0–1.5) | 1.44 (0.43–4.78) |  |
| Lump (mass) | 1 | 1 (1–1) | 1.33 (0.18–9.73) |  | 1 | 0 (0–0) | 3.35 (0.45–25.08) |  |
| Bleeding |  |  |  |  | 1 | 0 (0–0) | 3.35 (0.45–25.08) |  |
| Others |  |  |  |  | 3 | 0 (0–0) | 3.35 (0.99–11.35) |  |
| **First healthcare professional for evaluation** |  |  |  |  |  |  |  |  |
| Physician | 35 | 1 (0–2) | 1.01 (0.65–1.55) |  | 76 | 0 (0–3) | 1.00 | 0,903 |
| Dentist | 52 | 1 (0–3) | 1.00 | 0,973 | 19 | 0 (0–2.5) | 1.03 (0.62–1.71) |  |
| **First healthcare service sought** |  |  |  |  |  |  |  |  |
| **Physician** |  |  |  |  |  |  |  |  |
| Primary care center (public service) | 14 | 1 (0–2) | 1 (0.52–1.92) |  | 37 | 0 (0–2) | 1.00 | 0,737 |
| Specialized dental care center (secondary care, public service) | 1 | 1 (1–1) | 1.53 (0.21–11.41) |  | 6 | 0 (0–3.75) | 0.86 (0.36–2.06) |  |
| Hospital (tertiary care, public service) | 5 | 2 (0–4) | 1.08 (0.41–2.83) |  | 8 | 4 (0–6.5) | 0.69 (0.32–1.5) |  |
| Emergency room (public service) | 5 | 0 (0–1) | 1.55 (0.6–4.04) |  | 6 | 0.5 (0–1) | 1.57 (0.65–3.78) |  |
| Clinic (private service) | 8 | 1 (0–2.25) | 1.16 (0.53–2.56) |  | 17 | 0 (0–2) | 1.17 (0.65–2.09) |  |
| University dental clinc | 2 | 1 (0.5–1.5) | 1.67 (0.39–7.1) |  | 2 | 2 (1–3) | 0.99 (0.24–4.12) |  |
| **Dentist** |  |  |  |  |  |  |  |  |
| Primary care center (public service) | 13 | 1 (0–1) | 1.67 (0.85–3.28) |  | 9 | 0 (0–1) | 1.08 (0.52–2.25) |  |
| Specialized dental care center (secondary care, public service) | 1 | 3 (3–3) | 0.77 (0.1–5.68) |  |  |  |  |  |
| Hospital (tertiary care, public service) | 4 | 2 (0.75–4.5) | 0.82 (0.29–2.34) |  | 1 | 12 (12–12) | 0.36 (0.05–2.63) |  |
| Emergency room (public service) | 2 | 1 (1–1) | 1.53 (0.36–6.55) |  |  |  |  |  |
| Clinic (private service) | 29 | 1 (0–3) | 1.00 | 0,993 | 8 | 1 (0–2.25) | 1.15 (0.53–2.51) |  |
| University dental clinc | 3 | 0 (0–1) | 2.13 (0.64–7.11) |  | 1 | 0 (0–0) | 3.02 (0.41–22.33) |  |
| **Number of services visited until diagnosis** |  |  |  |  |  |  |  |  |
| 1 | 10 | 2 (1–3) | 0.94 (0.45–1.96) |  | 8 | 2.5 (0–8.75) | 0.54 (0.25–1.16) |  |
| 2 | 30 | 1 (0–3) | 1.00 | 0,874 | 24 | 0.5 (0–3.5) | 0.83 (0.5–1.38) |  |
| 3 | 25 | 0 (0–1) | 1.82 (1.04–3.19) |  | 40 | 0 (0–1.25) | 1.00 | 0,115 |
| 4 | 10 | 3 (0.25–3) | 0.85 (0.41–1.75) |  | 13 | 0 (0–1) | 1.16 (0.62–2.18) |  |
| 5 | 9 | 1 (0–2) | 1.22 (0.57–2.61) |  | 6 | 1.5 (0–13.5) | 0.4 (0.15–1.04) |  |
| 6 or more | 3 | 0 (0–3) | 1.28 (0.39–4.26) |  | 4 | 0 (0–0.25) | 1.8 (0.64–5.09) |  |
| **Professional delivering histopathological diagnosis** |  |  |  |  |  |  |  |  |
| Physician | 35 | 1 (0–2) | 0.84 (0.54–1.3) |  | 86 | 0 (0–2.75) | 1.00 | 0,497 |
| Dentist | 52 | 1 (0–3) | 1.00 | 0,435 | 9 | 1 (1–3) | 0.79 (0.39–1.57) |  |
| **Location of histopathological diagnosis** |  |  |  |  |  |  |  |  |
| **Physician** |  |  |  |  |  |  |  |  |
| Primary care center (public service) |  |  |  |  | 7 | 1 (0–4.5) | 0.8 (0.36–1.8) |  |
| Specialized dental care center (secondary care, public service) | 4 | 2 (0.75–5.75) | 0.55 (0.19–1.58) |  | 8 | 1 (0–4.25) | 0.74 (0.34–1.58) |  |
| Hospital (tertiary care, public service) | 20 | 1 (0–2) | 0.85 (0.47–1.53) |  | 39 | 0 (0–2) | 1.00 | 0,589 |
| Clinic (private service) | 1 | 1 (1–1) | 1.14 (0.15–8.45) |  | 14 | 0 (0–2.5) | 1.08 (0.58–2) |  |
| Hospital (private service) | 2 | 0 (0–0) | 3.6 (0.83–15.61) |  | 2 | 0 (0–0) | 2.85 (0.68–12.03) |  |
| University dental clinc | 8 | 2 (1–3.25) | 0.61 (0.28–1.36) |  | 16 | 0 (0–1.25) | 1.11 (0.62–2) |  |
| **Dentist** |  |  |  |  |  |  |  |  |
| Primary care center (public service) | 5 | 0 (0–3) | 1.15 (0.44–3) |  | 1 | 5 (5–5) | 0.51 (0.07–3.71) |  |
| Specialized dental care center (secondary care, public service) | 8 | 1 (0.75–3.75) | 0.75 (0.34–1.65) |  |  |  |  |  |
| Hospital (tertiary care, public service) | 6 | 3 (0.75–4.5) | 0.6 (0.25–1.46) |  | 3 | 1 (0.5–6.5) | 0.72 (0.22–2.33) |  |
| Clinic (private service) | 4 | 0.5 (0–1.5) | 1.25 (0.43–3.6) |  | 1 | 1 (1–1) | 1 (0.14–7.29) |  |
| Hospital (private service) | 2 | 0.5 (0.25–0.75) | 1.73 (0.41–7.36) |  |  |  |  |  |
| University dental clinc | 27 | 0 (0–2) | 1.00 | 0,267 | 4 | 2 (0.75–3) | 0.92 (0.33–2.59) |  |
