# Supplementary material for: An Analysis of the Timeline to Diagnosis and Treatment in Oral Cavity and Oropharynx Cancer
Source: Oral Dis. 2025 Dec 26;32(4):983–91. doi: 10.1111/odi.70171 (PMC13248584; doi:10.1111/odi.70171)
Supplement: Supplementary file 3 — Table S2: Health system diagnostic interval duration by sociodemographic and clinicopathological characteristics. [file ODI-32-983-s012.docx]

**Table S2.** Health system diagnostic interval duration by sociodemographic and clinicopathological characteristics.

| **Characteriscs** |  | **Oral cavity** |  |  |  | **Oropharynx** |  |  |
| --- | --- | --- | --- | --- | --- | --- | --- | --- |
|  | n | Median (IQR) | Hazard Ratio | P-value | n | Median (IQR) | Hazard Ratio | P-value |
| **Gender** |  |  |  |  |  |  |  |  |
| Male | 71 | 3 (1–5) | 1.00 | 0,189 | 76 | 3 (2–6.25) | 1.00 | 0,541 |
| Female | 16 | 5 (3–7.75) | 0.69 (0.4–1.2) |  | 19 | 4 (2–7.5) | 0.85 (0.51–1.42) |  |
| **Age at diagnosis** |  |  |  |  |  |  |  |  |
| ≤ 40 years | 5 | 3 (2–3) | 0.88 (0.35–2.23) |  | 2 | 10.5 (7.75–13.25) | 0.5 (0.12–2.07) |  |
| 41 - 60 years | 55 | 3 (1–5) | 1.00 | 0,785 | 61 | 3 (2–6) | 1.00 | 0,342 |
| > 60 years | 27 | 5 (2.5–9) | 0.68 (0.42–1.08) |  | 32 | 3 (1–6.25) | 1.04 (0.67–1.6) |  |
| **Ethnicity (self-reported)** |  |  |  |  |  |  |  |  |
| White | 33 | 3 (1–6) | 0.96 (0.61–1.53) |  | 33 | 3 (2–6) | 1.22 (0.78–1.91) |  |
| Black | 12 | 2.5 (1–7.5) | 1.03 (0.54–1.98) |  | 13 | 4 (2–5) | 1.33 (0.71–2.46) |  |
| Mixed | 42 | 4 (2–5.75) | 1.00 | 0,879 | 49 | 4 (2–9) | 1.00 | 0,378 |
| **Marital status** |  |  |  |  |  |  |  |  |
| Single | 22 | 3 (1–5) | 1.43 (0.85–2.42) |  | 29 | 4 (2–6) | 0.96 (0.6–1.55) |  |
| Married/living with a partner | 41 | 4 (2–6) | 1.00 | 0,178 | 45 | 3 (1–6) | 1.00 | 0,88 |
| Divorced/separated | 18 | 3 (1.25–6.75) | 0.91 (0.51–1.6) |  | 14 | 2 (2–4.75) | 1.38 (0.75–2.54) |  |
| Widowed | 6 | 5 (3.5–5.75) | 0.99 (0.42–2.35) |  | 7 | 9 (7.5–25) | 0.34 (0.15–0.77) |  |
| **Education** |  |  |  |  |  |  |  |  |
| < 1 year of schooling | 5 | 5 (3–16) | 0.45 (0.17–1.21) |  | 14 | 6 (4–11) | 0.57 (0.31–1.08) |  |
| 1 - 3 years of schooling | 18 | 4 (1.25–6) | 0.87 (0.48–1.58) |  | 12 | 3 (1–6.25) | 1.29 (0.66–2.52) |  |
| 4 - 7 years of schooling | 27 | 3 (2–5) | 1.00 | 0,114 | 34 | 3 (2–6) | 1.00 | 0,084 |
| 8 - 10 years of schooling | 11 | 2 (1–4.5) | 1.08 (0.54–2.19) |  | 12 | 6.5 (3.75–13.5) | 0.55 (0.28–1.07) |  |
| 11 - 14 years of schooling | 18 | 3 (1–6) | 0.69 (0.37–1.3) |  | 12 | 3 (2.75–7.5) | 1.01 (0.52–1.98) |  |
| 15 years of schooling or more | 8 | 5 (3–8) | 0.61 (0.28–1.35) |  | 11 | 2 (1–2.5) | 2.36 (1.17–4.77) |  |
| **Monthly income** |  |  |  |  |  |  |  |  |
| ≤ 1 minimum wage | 40 | 4 (1.75–6) | 0.99 (0.65–1.52) |  | 58 | 4 (2–6.75) | 1.00 | 0,467 |
| > 1 minimum wage | 47 | 3 (1.5–5.5) | 1.00 | 0,969 | 37 | 3 (1–6) | 1.17 (0.77–1.77) |  |
| **Smoking** |  |  |  |  |  |  |  |  |
| Never | 18 | 3 (2–6) | 0.8 (0.47–1.37) |  | 8 | 5.5 (2.75–6.25) | 0.93 (0.45–1.92) |  |
| Yes / Former smoker | 69 | 4 (1–6) | 1.00 | 0,417 | 87 | 3 (2–6.5) | 1.00 | 0,845 |
| **Alcohol comsumption** |  |  |  |  |  |  |  |  |
| Never | 21 | 3 (1–5) | 1.08 (0.65–1.77) |  | 16 | 4.5 (2–7.5) | 0.95 (0.55–1.63) |  |
| Yes / Former drinker | 66 | 4 (2–6) | 1.00 | 0,774 | 79 | 3 (2–6) | 1.00 | 0,851 |
| **Specific location of tumor** |  |  |  |  |  |  |  |  |
| Tongue | 42 | 3 (1–6) | 1.00 | 0,432 |  |  |  |  |
| Floor of mouth | 14 | 4 (1.25–5.75) | 1.28 (0.69–2.38) |  |  |  |  |  |
| Hard palate | 5 | 5 (4–8) | 0.83 (0.33–2.12) |  |  |  |  |  |
| Retromolar area | 11 | 3 (2.5–5.5) | 1.18 (0.6–2.33) |  |  |  |  |  |
| Alveolar ridge | 6 | 3 (2.25–3.75) | 1.82 (0.75–4.38) |  |  |  |  |  |
| Gengiva | 3 | 3 (2.5–8.5) | 0.97 (0.3–3.14) |  |  |  |  |  |
| Buccal mucosa | 6 | 4 (1.5–5) | 0.71 (0.28–1.83) |  |  |  |  |  |
| **p16 status** |  |  |  |  |  |  |  |  |
| Negative | 19 | 5 (2–6) | 0.77 (0.45–1.3) |  | 72 | 3.5 (2–6.25) | 1.00 | 0,908 |
| Positive | 6 | 3.5 (3–8.5) | 0.75 (0.32–1.75) |  | 23 | 3 (2–7) | 1.03 (0.64–1.65) |  |
| Unknown | 62 | 3 (1–5) | 1.00 | 0,332 |  |  |  |  |
| **T – Tumor size** |  |  |  |  |  |  |  |  |
| T1 | 8 | 9.5 (5–17.5) | 0.28 (0.12–0.63) |  | 9 | 2 (2–4) | 1.26 (0.61–2.6) |  |
| T2 | 14 | 5.5 (3.25–7.75) | 0.42 (0.22–0.8) |  | 12 | 3 (2–6.25) | 0.93 (0.48–1.81) |  |
| T3 | 17 | 2 (1–4) | 1.02 (0.58–1.8) |  | 31 | 4 (2–6) | 1.03 (0.64–1.64) |  |
| T4 | 46 | 3 (1–5) | 1.00 | **0,002** | 41 | 4 (2–8) | 1.00 | 0,535 |
| Tx | 2 | 5 (5–5) | 0.69 (0.17–2.85) |  | 2 | 21.5 (11.75–31.25) | 0.19 (0.03–1.47) |  |
| **N – Lymph node involvement** |  |  |  |  |  |  |  |  |
| N0 | 28 | 4.5 (2.75–9.25) | 1.00 | **0,042** | 12 | 3.5 (1.75–7.5) | 0.88 (0.45–1.7) |  |
| N1 | 10 | 3.5 (1–5) | 2.19 (1.03–4.64) |  | 21 | 5 (2–9) | 0.61 (0.34–1.08) |  |
| N2 | 27 | 3 (1–5) | 1.56 (0.91–2.67) |  | 33 | 3 (2–6) | 1.00 | 0,699 |
| N3 | 22 | 3 (2–5.75) | 1.73 (0.97–3.09) |  | 29 | 3 (2–7) | 0.67 (0.39–1.13) |  |
| **M – distant metastasis** |  |  |  |  |  |  |  |  |
| M0 | 85 | 3 (1–6) | 1.00 | 0,943 | 92 | 3 (2–6.25) | 1.00 | 0,82 |
| M1 | 2 | 4.5 (4.25–4.75) | 1.05 (0.26–4.32) |  | 3 | 4 (2.5–6.5) | 1.14 (0.36–3.63) |  |
| **Clinical staging** |  |  |  |  |  |  |  |  |
| I | 7 | 10 (7–21) | 0.31 (0.13–0.72) |  | 5 | 2 (2–9) | 0.93 (0.37–2.34) |  |
| II | 7 | 6 (3–22) | 0.48 (0.21–1.08) |  | 9 | 3 (1–7) | 1.03 (0.5–2.1) |  |
| III | 14 | 2.5 (1.25–4.75) | 1.26 (0.7–2.27) |  | 24 | 3.5 (2–6) | 1.09 (0.68–1.77) |  |
| IV | 59 | 3 (1–5) | 1.00 | **0,007** | 57 | 4 (2–7) | 1.00 | 0,882 |
| **Location of first symptom** |  |  |  |  |  |  |  |  |
| Oral cavity | 72 | 3 (1–6) | 1.00 | 0,609 | 15 | 3 (1.5–6) | 1.39 (0.76–2.54) |  |
| Cervical region | 2 | 8 (6–10) | 0.69 (0.17–2.84) |  | 34 | 2.5 (2–5.75) | 1.59 (0.99–2.57) |  |
| Oropharynx | 5 | 3 (1–5) | 1.36 (0.55–3.4) |  | 38 | 5 (2–7.75) | 1.00 | 0,291 |
| Others | 8 | 3.5 (2.75–5) | 1.46 (0.69–3.08) |  | 8 | 3.5 (1.75–6.75) | 1.17 (0.54–2.53) |  |
| **First noticed symptom by topography** |  |  |  |  |  |  |  |  |
| **Oral cavity** |  |  |  |  |  |  |  |  |
| Ulcer (wound) | 43 | 3 (1–5) | 1.00 | 0,986 | 10 | 3.5 (1.25–8.25) | 1.4 (0.67–2.91) |  |
| Pain | 7 | 5 (3–5.5) | 0.99 (0.44–2.25) |  | 3 | 4 (3.5–5) | 1.9 (0.56–6.37) |  |
| Lump (mass) | 6 | 3 (1.5–4.5) | 1.45 (0.61–3.46) |  | 1 | 1 (1–1) | 17.74 (2.22–141.5) |  |
| Spot | 8 | 8 (1.75–45.75) | 0.42 (0.19–0.92) |  |  |  |  |  |
| Swelling | 3 | 6 (4–6.5) | 0.77 (0.24–2.5) |  | 1 | 3 (3–3) | 3.03 (0.4–22.86) |  |
| Bleeding | 2 | 3.5 (2.75–4.25) | 1.31 (0.31–5.5) |  |  |  |  |  |
| Others | 3 | 12 (9–14) | 0.4 (0.12–1.29) |  |  |  |  |  |
| **Oropharynx** |  |  |  |  |  |  |  |  |
| Pain | 4 | 4 (2.5–5.75) | 1.01 (0.36–2.83) |  | 29 | 6 (3–9) | 1.00 | 0,3 |
| Spot | 1 | 1 (1–1) | 5.62 (0.74–42.78) |  | 3 | 2 (1.5–2.5) | 5.3 (1.53–18.33) |  |
| Ulcer (wound) |  |  |  |  | 2 | 1.5 (1.25–1.75) | 8.5 (1.91–37.87) |  |
| Lump (mass) |  |  |  |  | 1 | 16 (16–16) | 0.43 (0.06–3.2) |  |
| Swelling |  |  |  |  | 1 | 5 (5–5) | 1.7 (0.23–12.69) |  |
| Others |  |  |  |  | 2 | 3 (3–3) | 3.03 (0.69–13.2) |  |
| **Cervical region** |  |  |  |  |  |  |  |  |
| Pain | 1 | 12 (12–12) | 0.38 (0.05–2.8) |  | 4 | 2 (2–2.25) | 4.61 (1.52–13.96) |  |
| Lump (mass) |  |  |  |  | 24 | 3 (1–6) | 1.74 (0.99–3.05) |  |
| Swelling |  |  |  |  | 6 | 3 (2–4.75) | 2.14 (0.87–5.26) |  |
| Others | 1 | 4 (4–4) | 1.2 (0.16–8.86) |  |  |  |  |  |
| **Others** |  |  |  |  |  |  |  |  |
| Pain | 7 | 3 (2.5–4.5) | 1.45 (0.64–3.29) |  | 3 | 6 (3.5–15.5) | 0.81 (0.24–2.69) |  |
| Lump (mass) | 1 | 5 (5–5) | 0.87 (0.12–6.35) |  | 1 | 2 (2–2) | 5.59 (0.73–42.75) |  |
| Bleeding |  |  |  |  | 1 | 9 (9–9) | 0.82 (0.11–6.07) |  |
| Others |  |  |  |  | 3 | 3 (2–3.5) | 3.53 (1.03–12.03) |  |
| **First healthcare professional for evaluation** |  |  |  |  |  |  |  |  |
| Physician | 35 | 4 (3–7) | 0.83 (0.54–1.28) |  | 76 | 3 (2–6) | 1.00 | 0,54 |
| Dentist | 52 | 3 (1–5.25) | 1.00 | 0,394 | 19 | 4 (2–8) | 0.85 (0.51–1.42) |  |
| **First healthcare service sought** |  |  |  |  |  |  |  |  |
| **Physician** |  |  |  |  |  |  |  |  |
| Primary care center (public service) | 14 | 4 (2.25–5) | 1.32 (0.68–2.56) |  | 37 | 5 (2–7) | 1.00 | 0,905 |
| Specialized dental care center (secondary care, public service) | 1 | 12 (12–12) | 0.48 (0.07–3.58) |  | 6 | 6 (3.25–8.75) | 0.95 (0.4–2.26) |  |
| Hospital (tertiary care, public service) | 5 | 1 (1–3) | 2.86 (1.07–7.61) |  | 8 | 2 (1.75–3) | 2.16 (0.99–4.72) |  |
| Emergency room (public service) | 5 | 6 (5–8) | 0.79 (0.3–2.08) |  | 6 | 4.5 (2.25–6) | 1.46 (0.61–3.5) |  |
| Clinic (private service) | 8 | 7 (3–19) | 0.58 (0.26–1.29) |  | 17 | 2 (1–3) | 2.04 (1.13–3.68) |  |
| University dental clinc | 2 | 2.5 (1.75–3.25) | 2.55 (0.59–10.96) |  | 2 | 1 (1–1) | 13.78 (2.99–63.42) |  |
| **Dentist** |  |  |  |  |  |  |  |  |
| Primary care center (public service) | 13 | 3 (2–5) | 1.63 (0.83–3.22) |  | 9 | 6 (3–7) | 1.15 (0.55–2.41) |  |
| Specialized dental care center (secondary care, public service) | 1 | 2 (2–2) | 3.63 (0.48–27.58) |  |  |  |  |  |
| Hospital (tertiary care, public service) | 4 | 2 (0.75–3.75) | 2.14 (0.74–6.21) |  | 1 | 1 (1–1) | 13.78 (1.75–108.31) |  |
| Emergency room (public service) | 2 | 1.5 (1.25–1.75) | 4.9 (1.11–21.62) |  |  |  |  |  |
| Clinic (private service) | 29 | 4 (1–6) | 1.00 | 0,405 | 8 | 3 (1–8.5) | 1.05 (0.48–2.29) |  |
| University dental clinc | 3 | 1 (0.5–35) | 0.7 (0.21–2.38) |  | 1 | 13 (13–13) | 0.48 (0.07–3.54) |  |
| **Number of services visited until diagnosis** |  |  |  |  |  |  |  |  |
| 1 | 10 | 1 (0.25–1.75) | 6.27 (2.82–13.94) |  | 8 | 1 (1–2.25) | 4.85 (2.15–10.92) |  |
| 2 | 30 | 4 (2–6) | 1.00 | **0** | 24 | 3 (2–6) | 1.56 (0.93–2.62) |  |
| 3 | 25 | 4 (2–6) | 1.27 (0.73–2.21) |  | 40 | 4 (2–8.25) | 1.00 | **0** |
| 4 | 10 | 3.5 (3–5) | 1.18 (0.56–2.46) |  | 13 | 4 (2–6) | 0.93 (0.5–1.74) |  |
| 5 | 9 | 10 (5–12) | 0.59 (0.28–1.27) |  | 6 | 3.5 (2–9.5) | 1 (0.42–2.38) |  |
| 6 or more | 3 | 2 (1.5–2.5) | 3.89 (1.13–13.39) |  | 4 | 8 (6.75–17) | 0.36 (0.11–1.17) |  |
| **Professional delivering histopathological diagnosis** |  |  |  |  |  |  |  |  |
| Physician | 35 | 4 (3–6) | 0.87 (0.56–1.34) |  | 86 | 4 (2–6.75) | 1.00 | 0,366 |
| Dentist | 52 | 3 (1–5.25) | 1.00 | 0,529 | 9 | 2 (1–5) | 1.38 (0.69–2.74) |  |
| **Location of histopathological diagnosis** |  |  |  |  |  |  |  |  |
| **Physician** |  |  |  |  |  |  |  |  |
| Primary care center (public service) |  |  |  |  | 7 | 5 (3.5–9) | 0.64 (0.27–1.53) |  |
| Specialized dental care center (secondary care, public service) | 4 | 5 (3.5–6.5) | 1.28 (0.44–3.74) |  | 8 | 2 (1.75–6.75) | 1.16 (0.54–2.49) |  |
| Hospital (tertiary care, public service) | 20 | 4.5 (2.75–6.25) | 1.3 (0.71–2.37) |  | 39 | 4 (3–6.5) | 1.00 | 0,315 |
| Clinic (private service) | 1 | 3 (3–3) | 2.7 (0.36–20.45) |  | 14 | 2 (1–4.5) | 1.92 (1.03–3.56) |  |
| Hospital (private service) | 2 | 6 (6–6) | 0.97 (0.23–4.15) |  | 2 | 2 (2–2) | 3.58 (0.84–15.3) |  |
| University dental clinc | 8 | 4 (2.5–5) | 1.27 (0.57–2.82) |  | 16 | 5 (1–10.25) | 1.02 (0.57–1.84) |  |
| **Dentist** |  |  |  |  |  |  |  |  |
| Primary care center (public service) | 5 | 3 (3–5) | 1.97 (0.73–5.26) |  | 1 | 7 (7–7) | 0.77 (0.11–5.66) |  |
| Specialized dental care center (secondary care, public service) | 8 | 2 (1–3.25) | 2.66 (1.17–6.06) |  |  |  |  |  |
| Hospital (tertiary care, public service) | 6 | 1.5 (1–4.25) | 3.03 (1.2–7.65) |  | 3 | 2 (1.5–2.5) | 3.44 (1.04–11.43) |  |
| Clinic (private service) | 4 | 2.5 (1.5–3.5) | 2.91 (0.98–8.62) |  | 1 | 2 (2–2) | 3.58 (0.48–26.65) |  |
| Hospital (private service) | 2 | 2 (1.5–2.5) | 4.13 (0.94–18.06) |  |  |  |  |  |
| University dental clinc | 27 | 3 (1.5–10) | 1.00 | 0,649 | 4 | 3 (1–7.5) | 1.13 (0.4–3.19) |  |
