# Supplementary material for: An Analysis of the Timeline to Diagnosis and Treatment in Oral Cavity and Oropharynx Cancer
Source: Oral Dis. 2025 Dec 26;32(4):983–91. doi: 10.1111/odi.70171 (PMC13248584; doi:10.1111/odi.70171)
Supplement: Supplementary file 4 — Table S3: Pretreatment interval duration by sociodemographic, clinicopathological, and diagnostic journey characteristics. [file ODI-32-983-s001.docx]

**Table S3.** Pretreatment interval duration by sociodemographic, clinicopathological, and diagnostic journey characteristics.

| **Characteriscs** | **Oral cavity** | | | | | **Oropharynx** | | | |
| --- | --- | --- | --- | --- | --- | --- | --- | --- | --- |
|  | n | Median (IQR) | Hazard Ratio | P-value | n | | Median (IQR) | Hazard Ratio | P-value |
| **Gender** |  |  |  |  |  | |  |  |  |
| Male | 70 | 3 (2–4) | 1.00 | 0.288 | 70 | | 3 (2.25–4) | 1.00 | 0.163 |
| Female | 16 | 3 (2–3) | 1.34 (0.78–2.32) |  | 18 | | 3 (2–4) | 1.45 (0.86–2.46) |  |
| **Age at diagnosis** |  |  |  |  |  | |  |  |  |
| ≤ 40 years | 5 | 3 (2–3) | 1.65 (0.65–4.15) |  | 2 | | 1.5 (0.75–2.25) | 3.28 (0.79–13.62) |  |
| 41 - 60 years | 55 | 3 (2–4) | 1.00 | 0.292 | 58 | | 3 (2–4) | 1.00 | 0.102 |
| > 60 years | 26 | 3.5 (3–5) | 0.7 (0.43–1.13) |  | 28 | | 3 (2–4) | 0.74 (0.46–1.2) |  |
| **Ethnicity (self-reported)** |  |  |  |  |  | |  |  |  |
| White | 33 | 3 (2–4) | 0.96 (0.61–1.53) |  | 31 | | 3 (2–4) | 1.02 (0.65–1.62) |  |
| Black | 12 | 3 (2.75–4.5) | 0.89 (0.47–1.7) |  | 11 | | 3 (3–4) | 0.95 (0.49–1.84) |  |
| Mixed | 41 | 3 (2–4) | 1.00 | 0.873 | 46 | | 3 (2–4) | 1.00 | 0.917 |
| **Marital status** |  |  |  |  |  | |  |  |  |
| Single | 22 | 3.5 (2.25–4) | 0.79 (0.47–1.34) |  | 28 | | 3 (2–4) | 1.36 (0.83–2.2) |  |
| Married/living with a partner | 41 | 3 (2–4) | 1.00 | 0.383 | 44 | | 3.5 (2.75–4) | 1.00 | 0.22 |
| Divorced/separated | 18 | 3 (2–4.75) | 0.8 (0.46–1.41) |  | 11 | | 3 (2.5–3.5) | 1.57 (0.8–3.07) |  |
| Widowed | 5 | 4 (3–7) | 0.32 (0.11–0.93) |  | 5 | | 4 (2–4) | 1.41 (0.55–3.6) |  |
| **Education** |  |  |  |  |  | |  |  |  |
| < 1 year of schooling | 5 | 2 (2–2) | 2.48 (0.93–6.61) |  | 14 | | 3 (2–3.75) | 1.77 (0.92–3.41) |  |
| 1 - 3 years of schooling | 18 | 3.5 (3–4.75) | 1.27 (0.68–2.37) |  | 12 | | 4 (3–4) | 1 (0.51–1.96) |  |
| 4 - 7 years of schooling | 26 | 3 (3–4.75) | 1.00 | 0.069 | 30 | | 3 (2.25–4) | 1.00 | 0.088 |
| 8 - 10 years of schooling | 11 | 3 (2–4) | 1.75 (0.84–3.65) |  | 12 | | 4 (2.75–5) | 0.74 (0.38–1.44) |  |
| 11 - 14 years of schooling | 18 | 3 (2–4) | 1.83 (0.97–3.44) |  | 10 | | 3 (2–3.75) | 1.51 (0.73–3.12) |  |
| 15 years of schooling or more | 8 | 3 (2–3.25) | 2 (0.88–4.53) |  | 10 | | 4 (2.25–4) | 1.14 (0.55–2.36) |  |
| **Monthly income** |  |  |  |  |  | |  |  |  |
| ≤ 1 minimum wage | 40 | 3 (3–4) | 1.17 (0.76–1.81) |  | 51 | | 3 (2–4) | 1.00 | 0.101 |
| > 1 minimum wage | 46 | 3 (2–4) | 1.00 | 0.473 | 37 | | 4 (3–4) | 0.7 (0.45–1.07) |  |
| **Smoking** |  |  |  |  |  | |  |  |  |
| Never | 18 | 3 (3–3.75) | 1.14 (0.67–1.92) |  | 8 | | 4 (3.5–5) | 0.68 (0.33–1.41) |  |
| Yes / Former smoker | 68 | 3 (2–4) | 1.00 | 0.628 | 80 | | 3 (2–4) | 1.00 | 0.298 |
| **Alcohol comsumption** |  |  |  |  |  | |  |  |  |
| Never | 21 | 3 (2–3) | 1.22 (0.74–2.01) |  | 16 | | 4 (2–4) | 0.97 (0.56–1.68) |  |
| Yes / Former drinker | 65 | 3 (2–4) | 1.00 | 0.439 | 72 | | 3 (2–4) | 1.00 | 0.919 |
| **Specific location of tumor** |  |  |  |  |  | |  |  |  |
| Tongue | 42 | 3 (2–4) | 1.00 | 0.427 |  | |  |  |  |
| Floor of mouth | 13 | 4 (3–5) | 0.78 (0.41–1.45) |  |  | |  |  |  |
| Hard palate | 5 | 4 (3–4) | 0.75 (0.29–1.89) |  |  | |  |  |  |
| Retromolar area | 11 | 3 (2–3) | 0.96 (0.48–1.96) |  |  | |  |  |  |
| Alveolar ridge | 6 | 3.5 (2.25–6.25) | 0.55 (0.23–1.31) |  |  | |  |  |  |
| Gengiva | 3 | 4 (3.5–5) | 0.65 (0.2–2.09) |  |  | |  |  |  |
| Buccal mucosa | 6 | 3.5 (2.25–4.75) | 0.94 (0.4–2.22) |  |  | |  |  |  |
| **p16 status** |  |  |  |  |  | |  |  |  |
| Negative | 19 | 4 (3–5) | 0.71 (0.42–1.19) |  | 66 | | 3 (2.25–4) | 1.00 | 0.235 |
| Positive | 6 | 2.5 (2–3.75) | 0.99 (0.43–2.31) |  | 22 | | 3 (2–4) | 1.34 (0.82–2.19) |  |
| Unknown | 61 | 3 (2–4) | 1.00 | 0.195 |  | |  |  |  |
| **T – Tumor size** |  |  |  |  |  | |  |  |  |
| T1 | 8 | 3 (2.75–5.25) | 0.86 (0.41–1.83) |  | 9 | | 3 (2–4) | 0.72 (0.34–1.51) |  |
| T2 | 14 | 3 (2–3) | 1.2 (0.64–2.23) |  | 12 | | 4 (3–4.25) | 0.63 (0.32–1.22) |  |
| T3 | 17 | 4 (3–5) | 0.88 (0.5–1.54) |  | 30 | | 3.5 (2–4) | 1.04 (0.63–1.71) |  |
| T4 | 45 | 3 (2–4) | 1.00 | 0.7 | 35 | | 3 (2–4) | 1.00 | 0.38 |
| Tx | 2 | 1.5 (1.25–1.75) | 9.31 (2.12–40.89) |  | 2 | | 2.5 (1.25–3.75) | 0.73 (0.18–3.08) |  |
| **N – Lymph node involvement** |  |  |  |  |  | |  |  |  |
| N0 | 28 | 3 (2–4) | 1.00 | **0.039** | 12 | | 4 (3–4) | 0.87 (0.44–1.7) |  |
| N1 | 10 | 3.5 (2–6.75) | 0.44 (0.2–0.96) |  | 21 | | 3 (2–4) | 1.01 (0.57–1.77) |  |
| N2 | 27 | 3 (3–4) | 0.76 (0.45–1.3) |  | 30 | | 3 (3–4) | 1.00 | 0.675 |
| N3 | 21 | 3 (2–4) | 0.76 (0.43–1.35) |  | 25 | | 3 (2–4) | 1.02 (0.6–1.74) |  |
| **M – distant metastasis** |  |  |  |  |  | |  |  |  |
| M0 | 84 | 3 (2–4) | 1.00 | 0.11 | 86 | | 3 (2–4) | 1.00 | 0.407 |
| M1 | 2 | 2 (1.5–2.5) | 3.18 (0.77–13.11) |  | 2 | | 3 (3–3) | 1.82 (0.44–7.52) |  |
| **Clinical staging** |  |  |  |  |  | |  |  |  |
| I | 7 | 3 (2.5–4) | 1.16 (0.53–2.55) |  | 5 | | 4 (3–4) | 0.95 (0.38–2.38) |  |
| II | 7 | 2 (2–3) | 2.4 (1.08–5.33) |  | 9 | | 4 (2–4) | 0.99 (0.49–2.02) |  |
| III | 14 | 3 (2–4.75) | 0.75 (0.41–1.39) |  | 23 | | 3 (2–4) | 1.23 (0.75–2.03) |  |
| IV | 58 | 3 (3–4) | 1.00 | 0.711 | 51 | | 3 (2–4) | 1.00 | 0.907 |
| **Location of first symptom** |  |  |  |  |  | |  |  |  |
| Oral cavity | 71 | 3 (2–4) | 1.00 | 0.938 | 15 | | 3 (2–4) | 0.86 (0.47–1.58) |  |
| Cervical region | 2 | 3.5 (3.25–3.75) | 1.06 (0.26–4.33) |  | 31 | | 3 (2.5–4) | 1.14 (0.7–1.85) |  |
| Oropharynx | 5 | 4 (3–6) | 0.61 (0.25–1.51) |  | 35 | | 3 (2–4) | 1.00 | 0.63 |
| Others | 8 | 3 (2.75–3.25) | 0.83 (0.38–1.84) |  | 7 | | 4 (3–4) | 1.07 (0.47–2.42) |  |
| **First noticed symptom by topography** |  |  |  |  |  | |  |  |  |
| **Oral cavity** |  |  |  |  |  | |  |  |  |
| Ulcer (wound) | 42 | 3 (2–4) | 1.00 | 0.073 | 10 | | 2.5 (2–3.75) | 1.45 (0.7–3.02) |  |
| Pain | 7 | 4 (4–5.5) | 0.47 (0.21–1.07) |  | 3 | | 4 (4–5.5) | 0.25 (0.06–1.02) |  |
| Lump (mass) | 6 | 2.5 (2–3) | 2.43 (1–5.9) |  | 1 | | 2 (2–2) | 5.69 (0.74–43.66) |  |
| Spot | 8 | 3 (2–3.25) | 1.25 (0.58–2.66) |  |  | |  |  |  |
| Swelling | 3 | 4 (3–4.5) | 0.87 (0.27–2.83) |  | 1 | | 6 (6–6) | 0.22 (0.03–1.66) |  |
| Bleeding | 2 | 4.5 (3.25–5.75) | 0.48 (0.11–2.02) |  |  | |  |  |  |
| Others | 3 | 3 (3–4.5) | 0.77 (0.24–2.49) |  |  | |  |  |  |
| **Oropharynx** |  |  |  |  |  | |  |  |  |
| Pain | 4 | 3.5 (3–4.5) | 0.77 (0.27–2.14) |  | 26 | | 3.5 (2–4) | 1.00 | **0.053** |
| Spot | 1 | 7 (7–7) | 0.25 (0.03–1.85) |  | 2 | | 4 (2.5–5.5) | 0.21 (0.04–1.17) |  |
| Ulcer (wound) |  |  |  |  | 1 | | 3 (3–3) | 1.96 (0.26–14.65) |  |
| Lump (mass) |  |  |  |  | 3 | | 3 (3–3.5) | 1.32 (0.4–4.39) |  |
| Swelling |  |  |  |  | 1 | | 5 (5–5) | 0.38 (0.05–2.83) |  |
| Others |  |  |  |  | 2 | | 2.5 (2.25–2.75) | 2.91 (0.68–12.56) |  |
| **Cervical region** |  |  |  |  |  | |  |  |  |
| Pain | 1 | 3 (3–3) | 1.58 (0.22–11.63) |  | 4 | | 3 (2.75–3.5) | 1.05 (0.36–3.02) |  |
| Lump (mass) |  |  |  |  | 22 | | 3 (2.25–4) | 1.12 (0.63–1.99) |  |
| Swelling |  |  |  |  | 5 | | 3 (3–4) | 1.02 (0.39–2.66) |  |
| Others | 1 | 4 (4–4) | 0.76 (0.1–5.54) |  |  | |  |  |  |
| **Others** |  |  |  |  |  | |  |  |  |
| Pain | 7 | 3 (3–3.5) | 0.62 (0.26–1.52) |  | 2 | | 4 (4–4) | 0.8 (0.19–3.38) |  |
| Lump (mass) | 1 | 1 (1–1) | 36.93 (3.9–349.34) |  | 1 | | 3 (3–3) | 1.96 (0.26–14.65) |  |
| Bleeding |  |  |  |  | 1 | | 4 (4–4) | 0.8 (0.11–5.9) |  |
| Others |  |  |  |  | 3 | | 3 (3–3.5) | 1.32 (0.4–4.39) |  |
| **First healthcare professional for evaluation** |  |  |  |  |  | |  |  |  |
| Physician | 35 | 3 (3–4) | 1.14 (0.73–1.76) |  | 70 | | 3 (2–4) | 1.00 | 0.385 |
| Dentist | 51 | 3 (2–5) | 1.00 | 0.564 | 18 | | 4 (2.25–4) | 0.79 (0.47–1.34) |  |
| **First healthcare service sought by professional** |  |  |  |  |  | |  |  |  |
| **Physician** |  |  |  |  |  | |  |  |  |
| Primary care center (public service) | 14 | 3 (3–4) | 1.21 (0.63–2.32) |  | 35 | | 3 (2–4) | 1.00 | 0.358 |
| Specialized dental care center (secondary care, public service) | 1 | 3 (3–3) | 1.97 (0.26–14.7) |  | 5 | | 4 (3–4) | 0.64 (0.25–1.65) |  |
| Hospital (tertiary care, public service) | 5 | 4 (2–4) | 1.14 (0.44–2.98) |  | 8 | | 3 (2–3) | 1.89 (0.86–4.15) |  |
| Emergency room (public service) | 5 | 4 (3–4) | 1.39 (0.53–3.66) |  | 4 | | 3.5 (3–4.25) | 0.76 (0.27–2.14) |  |
| Clinic (private service) | 8 | 3 (3–3) | 1.18 (0.53–2.62) |  | 16 | | 3 (2.75–4) | 1.02 (0.57–1.85) |  |
| University dental clinc | 2 | 3 (2.5–3.5) | 1.69 (0.4–7.17) |  | 2 | | 4.5 (3.25–5.75) | 0.27 (0.06–1.29) |  |
| **Dentist** |  |  |  |  |  | |  |  |  |
| Primary care center (public service) | 12 | 3 (3–3.25) | 1.23 (0.62–2.43) |  | 9 | | 4 (3–5) | 0.53 (0.25–1.16) |  |
| Specialized dental care center (secondary care, public service) | 1 | 6 (6–6) | 0.5 (0.07–3.69) |  |  | |  |  |  |
| Hospital (tertiary care, public service) | 4 | 3 (1.75–4.25) | 1.49 (0.52–4.27) |  | 1 | | 2 (2–2) | 5.05 (0.67–38.29) |  |
| Emergency room (public service) | 2 | 4 (2.5–5.5) | 0.69 (0.16–2.94) |  |  | |  |  |  |
| Clinic (private service) | 29 | 3 (2–5) | 1.00 | 0.565 | 7 | | 4 (3–4) | 0.99 (0.44–2.25) |  |
| University dental clinc | 3 | 2 (2–2.5) | 3.61 (1.06–12.23) |  | 1 | | 4 (4–4) | 0.73 (0.1–5.38) |  |
| **Number of services visited until diagnosis** |  |  |  |  |  | |  |  |  |
| 1 | 10 | 3 (2–4.75) | 0.79 (0.38–1.65) |  | 7 | | 3 (3–4) | 0.78 (0.34–1.8) |  |
| 2 | 30 | 3 (2.25–4) | 1.00 | 0.539 | 22 | | 3 (3–4) | 1.12 (0.66–1.9) |  |
| 3 | 25 | 3 (2–4) | 0.87 (0.5–1.5) |  | 37 | | 4 (2–4) | 1.00 | 0.566 |
| 4 | 9 | 4 (3–6) | 0.63 (0.3–1.33) |  | 12 | | 3 (2.75–4) | 1.06 (0.54–2.07) |  |
| 5 | 9 | 3 (3–4) | 1.19 (0.56–2.53) |  | 6 | | 3 (3–3.75) | 1.11 (0.47–2.63) |  |
| 6 or more | 3 | 2 (1.5–4.5) | 0.77 (0.23–2.59) |  | 4 | | 3 (2–4.25) | 1.01 (0.36–2.83) |  |
| **Professional delivering histopathological diagnosis** |  |  |  |  |  | |  |  |  |
| Physician | 35 | 3 (3–4) | 0.86 (0.55–1.33) |  | 80 | | 3 (2–4) | 1.00 | 0.872 |
| Dentist | 51 | 3 (2–4) | 1.00 | 0.485 | 8 | | 3.5 (2.75–4) | 1.06 (0.51–2.2) |  |
| **Location of histopathological diagnosis by professional** |  |  |  |  |  | |  |  |  |
| **Physician** |  |  |  |  |  | |  |  |  |
| Primary care center (public service) |  |  |  |  | 7 | | 4 (3–4.5) | 0.68 (0.3–1.54) |  |
| Specialized dental care center (secondary care, public service) | 4 | 3 (2.75–3) | 1.69 (0.58–4.91) |  | 5 | | 3 (3–4) | 0.9 (0.35–2.29) |  |
| Hospital (tertiary care, public service) | 20 | 3 (3–5) | 0.57 (0.31–1.04) |  | 38 | | 3 (2–4) | 1.00 | 0.357 |
| Clinic (private service) | 1 | 8 (8–8) | 0.13 (0.02–1.07) |  | 13 | | 3 (3–4) | 0.95 (0.51–1.8) |  |
| Hospital (private service) | 2 | 3.5 (3.25–3.75) | 0.93 (0.22–3.91) |  | 2 | | 3 (2.5–3.5) | 1.2 (0.29–4.98) |  |
| University dental clinc | 8 | 3 (2–4) | 1.24 (0.56–2.75) |  | 15 | | 3 (3–4.5) | 0.63 (0.35–1.16) |  |
| **Dentist** |  |  |  |  |  | |  |  |  |
| Primary care center (public service) | 4 | 4.5 (3–6.25) | 0.42 (0.14–1.23) |  | 1 | | 4 (4–4) | 0.69 (0.09–5.04) |  |
| Specialized dental care center (secondary care, public service) | 8 | 3 (2–6.25) | 0.53 (0.23–1.2) |  |  | |  |  |  |
| Hospital (tertiary care, public service) | 6 | 3 (2–4) | 1.24 (0.51–3.03) |  | 2 | | 2.5 (2.25–2.75) | 2.37 (0.56–9.99) |  |
| Clinic (private service) | 4 | 3.5 (3–4.25) | 0.79 (0.28–2.27) |  | 1 | | 4 (4–4) | 0.69 (0.09–5.04) |  |
| Hospital (private service) | 2 | 3 (2.5–3.5) | 1.19 (0.28–5.05) |  |  | |  |  |  |
| University dental clinc | 27 | 3 (2–4) | 1.00 | 0.335 | 4 | | 3.5 (2.5–4.25) | 0.81 (0.29–2.28) |  |
