# Supplementary material for: An Analysis of the Timeline to Diagnosis and Treatment in Oral Cavity and Oropharynx Cancer
Source: Oral Dis. 2025 Dec 26;32(4):983–91. doi: 10.1111/odi.70171 (PMC13248584; doi:10.1111/odi.70171)
Supplement: Supplementary file 5 — Table S4: Negative binomial regression model of the patient interval in oral cavity cancer patients. [file ODI-32-983-s010.docx]

**Table S4.** Negative binomial regression model of the patient interval in oral cavity cancer patients.

| **Variable** | **IRR (IC95%)** | **Standard Error** | **p-value** |
| --- | --- | --- | --- |
| Intercept | 4.76 (1.71–13.25) | 0,5222 | 0.0028 ** |
| **Race** |  |  |  |
| Black | 0.23 (0.08– 0.73) | 0,5762 | 0.0120 * |
| Mixed | 0.60 (0.30– 1.21) | 0,3548 | 0.1542 |
| **N – Lymph node involvement** |  |  |  |
| N1 | 0.23 (0.07– 0.81) | 0,6386 | 0.0220 * |
| N2 | 1.34 (0.63– 2.86) | 0,3871 | 0.4528 |
| N3 | 0.61 (0.26– 1.41) | 0,4272 | 0.2485 |
| **Number of services visited until diagnosis** |  |  |  |
| 2 | 1.12 (0.39– 3.22) | 0,5384 | 0.8342 |
| 3 | 0.32 (0.11– 0.96) | 0,5613 | 0.0422 * |
| 4 | 1.23 (0.38– 4.01) | 0,6036 | 0.7328 |
| 5 | 0.54 (0.15– 1.99) | 0,6662 | 0.3532 |
| 6 | 0.63 (0.08– 4.81) | 1,0379 | 0.6552 |

Statistical significance is indicated by the following codes: ** p < 0.01; * p < 0.05; no marking indicates p ≥ 0.1 (not significant).
