# Supplementary material for: An Analysis of the Timeline to Diagnosis and Treatment in Oral Cavity and Oropharynx Cancer
Source: Oral Dis. 2025 Dec 26;32(4):983–91. doi: 10.1111/odi.70171 (PMC13248584; doi:10.1111/odi.70171)
Supplement: Supplementary file 6 — Table S5: Negative binomial regression model of the patient interval in oropharynx cancer patients. [file ODI-32-983-s004.docx]

**Table S5.** Negative binomial regression model of the patient interval in oropharynx cancer patients.

| **Variable** | **IRR (IC95%)** | **Standard Error** | **p-value** |
| --- | --- | --- | --- |
| Intercept | 8.26 (2.32–29.43) | 0,65 | 0.0011 ** |
| **Montly icome** |  |  |  |
| > 1 minimum wage | 0.42 (0.19– 0.97) | 0,42 | 0.0420 * |
| **Race** |  |  |  |
| Black | 0.95 (0.32– 2.88) | 0,56 | 0.9337 |
| Mixed | 2.25 (1.02– 4.99) | 0,41 | 0.0458 * |
| **HPV status** |  |  |  |
| Positive | 2.38 (1.00– 5.67) | 0,44 | 0.0499 * |
| **Number of services visited until diagnosis** |  |  |  |
| 2 | 0.38 (0.11– 1.35) | 0,65 | 0.1338 |
| 3 | 0.17 (0.05– 0.61) | 0,65 | 0.0065 ** |
| 4 | 0.12 (0.02– 0.57) | 0,81 | 0.0080 ** |
| 5 | 0.98 (0.17– 5.66) | 0,90 | 0.9798 |
| 6 | 0.04 (0.00– 0.70) | 1,41 | 0.0266 * |

Statistical significance is indicated by the following codes: ** p < 0.01; * p < 0.05; no marking indicates p ≥ 0.1 (not significant).
