# Supplementary material for: An Analysis of the Timeline to Diagnosis and Treatment in Oral Cavity and Oropharynx Cancer
Source: Oral Dis. 2025 Dec 26;32(4):983–91. doi: 10.1111/odi.70171 (PMC13248584; doi:10.1111/odi.70171)
Supplement: Supplementary file 7 — Table S6: Negative binomial regression model of the health system diagnostic interval in oral cavity cancer patients. [file ODI-32-983-s005.docx]

**Table S6.** Negative binomial regression model of the health system diagnostic interval in oral cavity cancer patients.

| **Variable** | **IRR (IC95%)** | **Standard Error** | **p-value** |
| --- | --- | --- | --- |
| Intercept | 7.75 (1.84–32.63) | 0,7333 | 0.0052 ** |
| **Marital status** |  |  |  |
| Married/living with a partner | 1.24 (0.71– 2.15) | 0,2815 | 0.4509 |
| Divirced/separated | 1.78 (0.88– 3.61) | 0,3602 | 0.1083 |
| Widowed | 0.99 (0.38– 2.56) | 0,4874 | 0.9754 |
| **Montly icome** |  |  |  |
| > 1 minimum wage | 0.86 (0.53– 1.39) | 0,2469 | 0.5384 |
| **Etilism** |  |  |  |
| Yes/Former drinker | 0.59 (0.36– 0.99) | 0,2601 | 0.0450 * |
| **HPV status** |  |  |  |
| Positive | 0.72 (0.26– 1.99) | 0,5219 | 0.5221 |
| Negative | 0.82 (0.47– 1.42) | 0,2823 | 0.4755 |
| **Clinical staging** |  |  |  |
| II | 0.86 (0.29– 2.55) | 0,5546 | 0.7853 |
| III | 0.18 (0.07– 0.47) | 0,4852 | <0.001 *** |
| IV | 0.22 (0.10– 0.52) | 0,4327 | <0.001 *** |
| **Number of services visited until diagnosis** |  |  |  |
| 2 | 6.12 (2.47–15.18) | 0,4631 | <0.001 *** |
| 3 | 4.11 (1.60–10.56) | 0,4815 | 0.0033 ** |
| 4 | 4.34 (1.54–12.22) | 0,5283 | 0.0055 ** |
| 5 | 10.92 (3.69–32.31) | 0,5533 | <0.001 *** |
| 6 | 2.97 (0.62–14.30) | 0,8018 | 0.1746 |
| **First healthcare professional for evaluation** |  |  |  |
| Dentist | 0.89 (0.54– 1.47) | 0,2556 | 0.6414 |
| **Professional delivering histopathological diagnosis** |  |  |  |
| Dentist | 0.81 (0.49– 1.34) | 0,2559 | 0.4147 |

Statistical significance is indicated by the following codes: *** p < 0,001; ** p < 0.01; * p < 0.05; no marking indicates p ≥ 0.1 (not significant).
