# Supplementary material for: An Analysis of the Timeline to Diagnosis and Treatment in Oral Cavity and Oropharynx Cancer
Source: Oral Dis. 2025 Dec 26;32(4):983–91. doi: 10.1111/odi.70171 (PMC13248584; doi:10.1111/odi.70171)
Supplement: Supplementary file 8 — Table S7: Negative binomial regression model of the health system diagnostic interval in oropharynx cancer patients. [file ODI-32-983-s011.docx]

**Table S7.** Negative binomial regression model of the health system diagnostic interval in oropharynx cancer patients.

| **Variable** | **IRR (IC95%)** | **Standard Error** | **p-value** |
| --- | --- | --- | --- |
| Intercept | 2.76 (1.20– 6.14) | 0,41 | 0.0129 * |
| **Marital status** |  |  |  |
| Married/living with a partner | 0.83 (0.56– 1.21) | 0,19 | 0.3047 |
| Divorced/separated | 0.66 (0.39– 1.10) | 0,26 | 0.1123 |
| Windowed | 2.40 (1.39– 4.26) | 0,29 | 0.0021 ** |
| **Education** |  |  |  |
| 1 – 3 years of schooling | 0.43 (0.23– 0.81) | 0,32 | 0.0090 ** |
| 4 – 7 years of schooling | 0.53 (0.33– 0.85) | 0,24 | 0.0076 ** |
| 8 – 10 years of schooling | 0.83 (0.48– 1.43) | 0,29 | 0.5050 |
| 11 – 14 years of schooling | 0.63 (0.34– 1.18) | 0,31 | 0.1420 |
| 15 years of schooling or more | 0.28 (0.14– 0.55) | 0,35 | <0.001 *** |
| **Montly icome** |  |  |  |
| > 1 minimum wage | 1.31 (0.90– 1.93) | 0,18 | 0.1367 |
| **Number of services visited until diagnosis** |  |  |  |
| 2 | 2.55 (1.21– 5.60) | 0,38 | 0.0142 * |
| 3 | 3.29 (1.61– 7.00) | 0,37 | 0.0012 ** |
| 4 | 3.17 (1.46– 7.16) | 0,40 | 0.0038 ** |
| 5 | 3.26 (1.33– 8.29) | 0,46 | 0.0109 * |
| 6 | 8.23 (3.35–21.46) | 0,47 | <0.001 *** |

Statistical significance is indicated by the following codes: *** p < 0,001; ** p < 0.01; * p < 0.05; no marking indicates p ≥ 0.1 (not significant).
