# Supplementary material for: An Analysis of the Timeline to Diagnosis and Treatment in Oral Cavity and Oropharynx Cancer
Source: Oral Dis. 2025 Dec 26;32(4):983–91. doi: 10.1111/odi.70171 (PMC13248584; doi:10.1111/odi.70171)
Supplement: Supplementary file 9 — Table S8: Negative binomial regression model of the pretreatment interval in oral cavity cancer patients. [file ODI-32-983-s006.docx]

**Table S8.** Negative binomial regression model of the pretreatment interval in oral cavity cancer patients.

| **Variable** | **IRR (IC95%)** | **Standard Error** | **p-value** |
| --- | --- | --- | --- |
| Intercept | 1.61 (0.75–3.46) | 0,3900 | 0.2221 |
| **Sex** |  |  |  |
| Female | 1.30 (0.89–1.90) | 0,1929 | 0.1743 |
| **Education** |  |  |  |
| 1-3 years of schooling | 1.57 (0.85–2.90) | 0,3132 | 0.1494 |
| 4-7 years of schooling | 1.68 (0.90–3.12) | 0,3161 | 0.1015 |
| 8-10 years of schooling | 1.60 (0.80–3.17) | 0,3502 | 0.1822 |
| 11-14 years of schooling | 1.05 (0.55–2.02) | 0,3317 | 0.8735 |
| 15 years of schooling or more | 1.48 (0.70–3.12) | 0,3808 | 0.3031 |
| **Marital status** |  |  |  |
| Married/living with a partner | 0.89 (0.65–1.22) | 0,1619 | 0.4679 |
| Divorced/separated | 1.03 (0.70–1.51) | 0,1968 | 0.8906 |
| Widowed | 2.35 (1.41–3.92) | 0,2607 | 0.0010 ** |
| **p16 status** |  |  |  |
| Positive | 0.98 (0.55–1.74) | 0,2915 | 0.9472 |
| Negative | 0.95 (0.70–1.29) | 0,1546 | 0.7491 |
| **Specific location of primary tumor** |  |  |  |
| Floor of mouth | 1.13 (0.77–1.67) | 0,1984 | 0.5336 |
| Hard palate | 1.34 (0.76–2.35) | 0,2867 | 0.3082 |
| Retromolar area | 1.08 (0.74–1.57) | 0,1939 | 0.7030 |
| Alveolar ridge | 0.87 (0.50–1.50) | 0,2812 | 0.6109 |
| Gingiva | 1.27 (0.66–2.47) | 0,3379 | 0.4725 |
| Buccal mucosa | 1.21 (0.74–1.98) | 0,2516 | 0.4461 |
| **T – Tumor size** |  |  |  |
| N1 | 1.50 (0.99–2.27) | 0,2109 | 0.0550 . |
| N2 | 1.08 (0.76–1.55) | 0,1817 | 0.6551 |
| N3 | 1.27 (0.89–1.82) | 0,1828 | 0.1896 |
| **Alcohol consumption** |  |  |  |
| Yes / Former drinker | 1.08 (0.74–1.59) | 0,1959 | 0.6901 |
| **M – distant metastasis** |  |  |  |
| M1 | 0.45 (0.16–1.32) | 0,5457 | 0.1455 |

Statistical significance is indicated by the following codes: ** p < 0.01; no marking indicates p ≥ 0.1 (not significant).
