# Supplementary material for: An Analysis of the Timeline to Diagnosis and Treatment in Oral Cavity and Oropharynx Cancer
Source: Oral Dis. 2025 Dec 26;32(4):983–91. doi: 10.1111/odi.70171 (PMC13248584; doi:10.1111/odi.70171)
Supplement: Supplementary file 10 — Table S9: Negative binomial regression model of the pretreatment interval in oropharynx cancer patients. [file ODI-32-983-s003.docx]

**Table S9.** Negative binomial regression model of the pretreatment interval in oropharynx cancer patients.

| **Variable** | **IRR (IC95%)** | **Standard Error** | **p-value** |
| --- | --- | --- | --- |
| (Intercept) | 1.25 (0.27– 5.69) | 0,77 | 0.7761 |
| **Sex** |  |  |  |
| Female | 1.15 (0.79– 1.70) | 0,20 | 0.4637 |
| **Age** |  |  |  |
| 41-60 years | 2.85 (0.85– 9.56) | 0,62 | 0.0903 . |
| >60 years | 3.02 (0.90–10.08) | 0,61 | 0.0722 . |
| **Education** |  |  |  |
| 1-3 years of schooling | 1.10 (0.66– 1.81) | 0,26 | 0.7221 |
| 4-7 years of schooling | 1.16 (0.76– 1.76) | 0,22 | 0.5039 |
| 8-10 years of schooling | 1.42 (0.87– 2.33) | 0,25 | 0.1570 |
| 11-14 years of schooling | 0.87 (0.49– 1.55) | 0,29 | 0.6406 |
| 15 years of schooling or more | 0.87 (0.49– 1.57) | 0,30 | 0.6530 |
| **Monthly income** |  |  |  |
| > 1 minimum wage | 1.18 (0.87– 1.61) | 0,16 | 0.2894 |
| **Smoking** |  |  |  |
| Yes / Former smoker | 0.85 (0.47– 1.53) | 0,30 | 0.5902 |
| **Alcohol consumption** |  |  |  |
| Yes / Former smoker | 0.99 (0.60– 1.62) | 0,25 | 0.9599 |
| **N – Lymph node involvement** |  |  |  |
| N1 | 0.98 (0.63– 1.52) | 0,23 | 0.9176 |
| N2 | 0.92 (0.62– 1.37) | 0,20 | 0.6911 |
| N3 | 0.83 (0.54– 1.28) | 0,22 | 0.3948 |
| **Location of histopathological diagnosis by professional** |  |  |  |
| *Physician* |  |  |  |
| Specialized dental care center (secondary care, public service) |  |  |  |
| Specialized dental care center (secondary care, public service) | 0.73 (0.37– 1.46) | 0,35 | 0.3803 |
| Hospital (tertiary care, public service) | 0.85 (0.53– 1.36) | 0,24 | 0.4993 |
| Clinic (private service) | 0.95 (0.53– 1.70) | 0,30 | 0.8571 |
| Hospital (private service) | 0.96 (0.36– 2.56) | 0,50 | 0.9429 |
| University dental clinic | 1.00 (0.56– 1.79) | 0,30 | 0.9908 |
| *Dentistry* |  |  |  |
| Primary care center (public service) | 1.35 (0.39– 4.68) | 0,63 | 0.6367 |
| Hospital (tertiary care, public service) | 0.60 (0.20– 1.78) | 0,56 | 0.3538 |
| Clinic (private service) | 1.02 (0.30– 3.53) | 0,63 | 0.9714 |
| University dental clinic | 0.74 (0.34– 1.58) | 0,39 | 0.4307 |

Statistical significance is indicated by the following codes: no marking indicates p ≥ 0.1 (not significant).
