# Supplementary material for: An Analysis of the Timeline to Diagnosis and Treatment in Oral Cavity and Oropharynx Cancer
Source: Oral Dis. 2025 Dec 26;32(4):983–91. doi: 10.1111/odi.70171 (PMC13248584; doi:10.1111/odi.70171)
Supplement: Supplementary file 11 — Table S10: Sociodemographic and clinicopathological characteristics of patients by primary tumor site. [file ODI-32-983-s002.docx]

**Table S10.** Sociodemographic and clinicopathological characteristics of patients by primary tumor site.

|  | **Total** | | **Oral Cavity** | | **Oropharynx** | |  | |
| --- | --- | --- | --- | --- | --- | --- | --- | --- |
| **Characteristics** | **n 182 (%)** | | **n 87 (%)** | | **n 95 (%)** | | **X²** | |
| **Gender** |  | |  | |  | |  | |
| Female | 35 (19.2) | | 16 (18.4) | | 19 (20.0) | | 0.076 | |
| Male | 147 (80.8) | | 71 (81.6) | | 76 (80.0) | | p = 0.783 | |
| **Age at diagnosis** |  | |  | |  | |  | |
| ≤40 years | 7 (3.8) | | 5 (5.7) | | 2 (2.1) | |  | |
| 41-60 years | 116 (63.7) | | 55 (63.2) | | 61 (64.2) | | 1.671 | |
| >60 years | 59 (32.4) | | 27 (31.0) | | 32 (33.7) | | p = 0.434 | |
| **Ethnicity (self-reported)** |  | |  | |  | |  | |
| White | 66 (36.3) | | 33 (37.9) | | 33 (34.7) | |  | |
| Black | 25 (13.7) | | 12 (13.8) | | 13 (13.7) | | 0.227 | |
| Mixed | 91 (50.0) | | 42 (48.3) | | 49 (51.6) | | p = 0.893 | |
| **Marital status** |  | |  | |  | |  | |
| Single | 51 (28.0) | | 22 (25.3) | | 29 (30.5) | |  | |
| Married/living with a partner | 86 (47.3) | | 41 (47.1) | | 45 (47.4) | |  | |
| Divorced/separated | 32 (17.6) | | 18 (20.7) | | 14 (14.7) | | 1.375 | |
| Widowed | 13 (7.1) | | 6 (6.9) | | 7 (7.4) | | p = 0.711 | |
| **Education** |  | |  | |  | |  | |
| < 1 year of schooling | 19 (10.4) | | 5 (5.7) | | 14 (14.7) | |  | |
| 1-3 years of schooling | 30 (16.5) | | 18 (20.7) | | 12 (12.6) | |  | |
| 4-7 years of schooling | 61 (33.5) | | 27 (31.0) | | 34 (35.8) | |  | |
| 8-10 years of schooling | 23 (12.6) | | 11 (12.6) | | 12 (12.6) | |  | |
| 11-14 years of schooling | 30 (16.5) | | 18 (20.7) | | 12 (12.6) | | 7.647 | |
| 15 years of schooling or more | 19 (10.4) | | 8 (9.2) | | 11 (11.6) | | p = 0.177 | |
| **Monthly income** |  | |  | |  | |  | |
| ≤ 1 minimum wage* | 98 (53.8) | | 40 (46.0) | | 58 (61.1) | | 4.135 | |
| > 1 minimum wage* | 84 (46.2) | | 47 (54.0) | | 37 (38.9) | | p = 0.042* | |
| **Smoking** |  | |  | |  | |  | |
| Never* | 26 (14.3) | | 18 (20.7) | | 8 (8.4) | | 5.582 | |
| Yes / Former smoker* | 156 (85.7) | | 69 (79.3) | | 87 (91.6) | | p = 0.018* | |
| **Smoking load (packs/year)** |  | |  | |  | |  | |
| 0.1 to 5 | 11 (7.1) | | 5 (7.2) | | 6 (6.9) | |  | |
| 5.1 to 11.9 | 13 (8.3) | | 8 (11.6) | | 5 (5.7) | | 1.760 | |
| 12 or more | 132 (84.6) | | 56 (81.2) | | 76 (87.4) | | p = 0.415 | |
| **Alcohol consumption** |  | |  | |  | |  | |
| Never | 37 (20.3) | | 21 (24.1) | | 16 (16.8) | | 1.492 | |
| Yes / Former drinker | 145 (79.7) | | 66 (75.9) | | 79 (83.2) | | p = 0.222 | |
| **Specific location of primary tumor** |  | |  | |  | |  | |
| Tongue | 42 (23.1) | | 42 (48.3) | | - | |  | |
| Floor of mouth | 14 (7.7) | | 14 (16.1) | | - | |  | |
| Retromolar area | 11 (6.0) | | 11 (12.6) | | - | |  | |
| Alveolar ridge | 6 (3.3) | | 6 (6.9) | | - | |  | |
| Buccal mucosa | 6 (3.3) | | 6 (6.9) | | - | |  | |
| Hard palate | 5 (2.7) | | 5 (5.7) | | - | |  | |
| Gingiva | 3 (1.6) | | 3 (3.4) | | - | |  | |
| Oropharynx (base of the tongue, soft palate, tonsil) | 95 (52.2) | | - | | 95 (100) | | - | |
| **p16 status** |  | |  | |  | |  | |
| Positive* | 29 (15.9) | | 6 (6.9) | | 23 (24.2) | |  | |
| Negative* | 91 (50) | | 19 (21.8) | | 72 (75.8) | | 102.680 | |
| Unknown* | 62 (34.1) | | 62 (71.3) | | 0 (0.0) | | p <0.001* | |
| **T – Tumor size** |  | |  | |  | |  | |
| T1 | 17 (9.3) | | 8 (9.2) | | 9 (9.5) | |  | |
| T2 | 26 (14.3) | | 14 (16.1) | | 12 (12.6) | |  | |
| T3 | 48 (26.4) | | 17 (19.5) | | 31 (32.6) | |  | |
| T4 | 87 (47.8) | | 46 (52.9) | | 41 (43.2) | | 4.240 | |
| Tx | 4 (2.2) | | 2 (2.3) | | 2 (2.1) | | p = 0.375 | |
| **N – Lymph node involvement** |  | |  | |  | |  | |
| N0* | 40 (22.0) | | 28 (32.2) | | 12 (12.6) | |  | |
| N1 | 31 (17.0) | | 10 (11.5) | | 21 (22.1) | |  | |
| N2 | 60 (33.0) | | 27 (31.0) | | 33 (34.7) | | 11.535 | |
| N3 | 51 (28.0) | | 22 (25.3) | | 29 (30.5) | | p = 0.009* | |
| **M – distant metastasis** |  | |  | |  | |  | |
| M0 | 177 (97.3) | | 85 (97.7) | | 92 (96.8) | | 0.125 | |
| M1 | 5 (2.7) | | 2 (2.3) | | 3 (3.2) | | p = 0.723 | |
|  |  | |  | |  | |  | |
| **Clinical staging** |  | |  | |  | |  | |
| I | 12 (6.6) | | 7 (8.0) | | 5 (5.3) | |  | |
| II | 16 (8.8) | | 7 (8.0) | | 9 (9.5) | |  | |
| III | 38 (20.9) | | 14 (16.1) | | 24 (25.3) | | 2.903 | |
| IV | 116 (63.7) | 59 (67.8) | | 57 (60.0) | | p = 0.407 | |  |

*Chi-square test showed statistically significant difference between patients with tumors in oral cavity and oropharynx.
