# Supplementary material for: An Analysis of the Timeline to Diagnosis and Treatment in Oral Cavity and Oropharynx Cancer
Source: Oral Dis. 2025 Dec 26;32(4):983–91. doi: 10.1111/odi.70171 (PMC13248584; doi:10.1111/odi.70171)
Supplement: Supplementary file 12 — Table S11: Diagnostic itinerary characteristics by primary tumor site. [file ODI-32-983-s007.docx]

**Table S11.** Diagnostic itinerary characteristics by primary tumor site.

|  | **Total** | **Oral Cavity** | **Oropharynx** |  |
| --- | --- | --- | --- | --- |
| **Diagnostic itinerary** | **n 182 (%)** | **n 87 (%)** | **n 95 (%)** | **X²** |
| **First noticed symptom by topography** |  |  |  |  |
| **Oral cavity** |  |  |  |  |
| Pain | 10 (11.5) | 7 (9.7) | 3 (20.0) | 4.159  p = 0.655 |
| Ulcer (wound) | 53 (60.9) | 43 (59.7) | 10 (66.7) |  |
| Lump (mass) | 7 (8.0) | 6 (8.3) | 1 (6.7) |  |
| Spot | 8 (9.2) | 8 (11.1) | 0 (0.0) |  |
| Swelling | 4 (4.6) | 3 (4.2) | 1 (6.7) |  |
| Bleeding | 2 (2.3) | 2 (2.8) | 0 (0.0) |  |
| Others | 3 (3.4) | 3 (4.2) | 0 (0.0) |  |
| **Oropharynx** |  |  |  |  |
| Pain | 33 (76.7) | 4 (80) | 29 (76.3) |  |
| Ulcer (wound) | 2 (4.7) | 0 (0.0) | 2 (5.3) |  |
| Lump (mass) | 1 (2.3) | 0 (0.0) | 1 (2.6) |  |
| Spot | 4 (9.3) | 1 (20) | 3 (7.9) | 1.493  p = 0.914 |
| Swelling | 1 (2.3) | 0 (0.0) | 1 (2.6) |  |
| Others | 2 (4.7) | 0 (0.0) | 2 (5.3) |  |
| **Cervical** |  |  |  |  |
| Pain | 5 (13.9) | 1 (50.0) | 4 (11.8) |  |
| Lump (mass)* | 24 (66.7) | 0 (0.0) | 24 (70.6) | 20.753  p<0.001* |
| Swelling | 6 (16.7) | 0 (0.0) | 6 (17.6) |  |
| Others* | 1 (2.8) | 1 (50.0) | 0 (0.0) |  |
| **Others** |  |  |  |  |
| Pain | 10 (62.5) | 7 (87.5) | 3 (37.5) |  |
| Lump (mass) | 2 (12.5) | 1 (12.5) | 1 (12.5) | 5.600  p = 0.133 |
| Bleeding | 1 (6.3) | 0 (0.0) | 1 (12.5) |  |
| Others | 3 (18.8) | 0 (0.0) | 3 (37.5) |  |
| **Location of first symptom** |  |  |  |  |
| Oral cavity* | 87 (47.8) | 72 (82.8) | 15 (15.8) |  |
| Oropharynx* | 43 (23.6) | 5 (5.7) | 38 (40.0) |  |
| Cervical region* | 36 (19.8) | 2 (2.3) | 34 (35.8) | 90.939 |
| Others | 16 (8.8) | 8 (9.2) | 8 (8.4) | p <0.001* |
| **First healthcare service sought** |  |  |  |  |
| Primary care center (public service) | 73 (40.1) | 27 (31.0) | 46 (48.4) |  |
| Clinic (private service) | 62 (34.1) | 37 (42.5) | 25 (26.3) |  |
| Hospital (tertiary care, public service) | 18 (9.9) | 9 (10.3) | 9 (9.5) |  |
| Emergency room (public service) | 13 (7.1) | 7 (8.0) | 6 (6.3) | 9.511 |
| Specialized dental care center (secondary care, public service) | 8 (4.4) | 2 (2.3) | 6 (6.3) | p = 0.090 |
| University dental clinic | 8 (4.4) | 5 (5.7) | 3 (3.2) |  |
| **First healthcare professional for evaluation** |  |  |  |  |
| Physician* | 111 (61.0) | 35 (40.2) | 76 (80.0) | 30.189 |
| Dentist* | 71 (39.0) | 52 (59.8) | 19 (20.0) | p<0.001* |
| **Location of histopathological diagnosis** |  |  |  |  |
| Hospital (tertiary care, public service)* | 68 (37.4) | 26 (29.9) | 42 (44.2) |  |
| University dental clinic* | 55 (30.2) | 35 (40.2) | 20 (21.1) |  |
| Specialized dental care center (secondary care, public service) | 20 (11.0) | 12 (13.8) | 8 (8.4) |  |
| Clinic (private service)* | 20 (11.0) | 5 (5.7) | 15 (15.8) | 14.691 |
| Primary care center (public service) | 13 (7.1) | 5 (5.7) | 8 (8.4) | p = 0.012* |
| Hospital (private service) | 6 (3.3) | 4 (4.6) | 2 (2.1) |  |
| **Professional delivering the histopathological diagnosis** |  |  |  |  |
| Physician* | 121 (66.5) | 35 (40.2) | 86 (90.5) | 51.555 |
| Dentist* | 61 (33.5) | 52 (59.8) | 9 (9.5) | p<0.001 |
| **Number of services visited until histopathological diagnosis** |  |  |  |  |
| 1 | 18 (9.9) | 10 (11.5) | 8 (8.4) |  |
| 2 | 54 (26.7) | 30 (34.5) | 24 (25.3) |  |
| 3 | 65 (35.7) | 25 (28.7) | 40 (42.1) |  |
| 4 | 23 (12.6) | 10 (11.5) | 13 (13.7) |  |
| 5 | 15 (8.2) | 9 (10.3) | 6 (6.3) | 5.334 |
| 6 or more | 7 (3.8) | 3 (3.4) | 4 (4.2) | p = 0.502 |

*Chi-square test showed statistically significant difference between patients with tumors in oral cavity and oropharynx
