# Supplementary material for: An Analysis of the Timeline to Diagnosis and Treatment in Oral Cavity and Oropharynx Cancer
Source: Oral Dis. 2025 Dec 26;32(4):983–91. doi: 10.1111/odi.70171 (PMC13248584; doi:10.1111/odi.70171)
Supplement: Supplementary file 13 — Table S12: Clinical stage by race. [file ODI-32-983-s009.docx]

**Table S12.** Clinical Stage by Race

| **Clinical stage** | **Oral cavity** | | | |  | **Oropharynx** | | | |  |
| --- | --- | --- | --- | --- | --- | --- | --- | --- | --- | --- |
|  | **n (%)** | White  n (%) | Black  n (%) | Mixed  n (%) | **p -value** | **n (%)** | White  n (%) | Black  n (%) | Mixed  n (%) | **p -value** |
| I / II | 14 (16.1) | 6 (18.2) | 1 (8.3) | 7 (16.7) | 0.851  p = 0.652 | 14 (14.7) | 5 (15.2) | 3 (23.1) | 6 (12.2) | 0.615  p = 0.966 |
| III / IV | 73 (83.9) | 27 (81.8) | 11 (91.7) | 35 (83.3) |  | 81 (85.3) | 28 (84.8) | 10 (76.9) | 43 (87.8) |  |
